# Loss of lncRNA LINC01056 leads to sorafenib resistance in HCC

**DOI:** 10.1186/s12943-024-01988-y

**Published:** 2024-04-06

**Authors:** Yau-Tuen Chan, Junyu Wu, Yuanjun Lu, Qiucheng Li, Zixin Feng, Lin Xu, Hongchao Yuan, Tingyuan Xing, Cheng Zhang, Hor-Yue Tan, Yibin Feng, Ning Wang

**Affiliations:** 1 School of Chinese Medicine, The University of Hong Kong, Pok Fu Lam, Hong Kong; 2 Centre for Chinese Medicine New Drug Development, School of Chinese Medicine, Hong Kong Baptist University, Kowloon Tong, Hong Kong

**Keywords:** CRISPR/Cas9 screens, LINC01056, Sorafenib, Hepatocellular carcinoma, Fatty acid oxidation, PPARα

## Abstract

**Background and aims:**

Sorafenib is a major nonsurgical option for patients with advanced hepatocellular carcinoma (HCC); however, its clinical efficacy is largely undermined by the acquisition of resistance. The aim of this study was to identify the key lncRNA involved in the regulation of the sorafenib response in HCC.

**Materials and methods:**

A clustered regularly interspaced short palindromic repeats (CRISPR)/CRISPR-associated protein 9 (Cas9) single-guide RNA (sgRNA) synergistic activation mediator (SAM)-pooled lncRNA library was applied to screen for the key lncRNA regulated by sorafenib treatment. The role of the identified lncRNA in mediating the sorafenib response in HCC was examined in vitro and in vivo. The underlying mechanism was delineated by proteomic analysis. The clinical significance of the expression of the identified lncRNA was evaluated by multiplex immunostaining on a human HCC microtissue array.

**Results:**

CRISPR/Cas9 lncRNA library screening revealed that Linc01056 was among the most downregulated lncRNAs in sorafenib-resistant HCC cells. Knockdown of Linc01056 reduced the sensitivity of HCC cells to sorafenib, suppressing apoptosis in vitro and promoting tumour growth in mice in vivo. Proteomic analysis revealed that Linc01056 knockdown in sorafenib-treated HCC cells induced genes related to fatty acid oxidation (FAO) while repressing glycolysis-associated genes, leading to a metabolic switch favouring higher intracellular energy production. FAO inhibition in HCC cells with Linc01056 knockdown significantly restored sensitivity to sorafenib. Mechanistically, we determined that PPARα is the critical molecule governing the metabolic switch upon Linc01056 knockdown in HCC cells and indeed, PPARα inhibition restored the sorafenib response in HCC cells in vitro and HCC tumours in vivo. Clinically, Linc01056 expression predicted optimal overall and progression-free survival outcomes in HCC patients and predicted a better sorafenib response. Linc01056 expression indicated a low FAO level in HCC.

**Conclusion:**

Our study identified Linc01056 as a critical epigenetic regulator and potential therapeutic target in the regulation of the sorafenib response in HCC.

**Supplementary Information:**

The online version contains supplementary material available at 10.1186/s12943-024-01988-y.

## Introduction

Hepatocellular carcinoma (HCC) has historically been a serious healthcare problem worldwide, as it is the fifth most fatal cancer [1]. Major risk factors for HCC include hepatic viral infection, alcohol overuse, metabolic diseases and intake of aflatoxin B1 [2]. As HCC often causes only mild or no symptoms until advanced stages, patients are usually nonresponsive to curative treatments. For patients with early-stage HCC, surgery is the most promising option, resulting in a 5-year survival rate of > 70% [3]. Other treatments include liver transplantation and loco-regional therapies. However, only 40% of patients are diagnosed at early stages, leaving resection therapies impractical [4]. Sorafenib is the first FDA-approved first-line therapy for the systematic management of advanced and end-stage HCC. Sorafenib is a tyrosine kinase inhibitor that blocks the activity of enzymes essential for the growth and proliferation of HCC. It also exhibits antiangiogenic effects and extends overall survival in HCC patients [5]. However, only one-third of patients are responsive to sorafenib treatment, and relapse usually occurs within half a year [6]. The development of sorafenib resistance in HCC is the major challenge in treating patients with advanced-stage disease. The mechanisms underlying acquired sorafenib resistance are numerous and largely unknown, leaving a very large research gap and an urgent need for investigation.

Genetic variations and mutations are not only associated with cancer development but also play roles in developing drug resistance. Common sorafenib resistance mechanisms include changes in transporter proteins and drug targets [7, 8], as well as alterations in signalling pathways [9, 10]. Drug metabolism, autophagy, ferroptosis and modulation of cancer stem cells are also reported to be related to resistance. Many of these phenotypic changes are the result of epigenetic regulation. Emerging evidence has revealed that noncoding RNAs, including long noncoding RNAs (lncRNAs) and microRNAs (miRNAs), are important regulators of HCC biological processes. The roles of lncRNAs in cancer development have been shown to be diverse [6] and include sustaining proliferation, suppressing cell death and apoptosis, and promoting angiogenesis, migration and metastasis. LncRNAs also play important roles in metabolic reprogramming and immune evasion [11]. LncSNHG16 was reported to be upregulated in HCC cells exhibiting sorafenib resistance. LncSNHG16 is an endogenous sponge for miRNA-140-5p. In addition to functioning as miRNA sponges, lncRNAs can also act as RNA decoys and bind to transcription factors that inhibit their activities [12]. LncPANDA interacts with the transcription factor NF-YA to suppress gene expression and senescence acquisition [13]. Another lncRNA, LINC001134, can recruit SP1 to the p62 promoter, thus enhancing the activation of the antioxidative pathway and resulting in drug resistance in HCC [14]. The expression of lncRNAs can be induced by the action of miRNAs, and their subsequent nuclear translocation is induced by sorafenib. LncSNHG1 plays a role in resistance by activating the Akt signalling pathway [15]. Hence, searching for oncogenic lncRNAs is beneficial for identifying drug targets and improving current therapeutic options.

Due to the noncoding nature and diverse actions of lncRNAs, a systematic approach is needed to identify their functional roles in HCC drug resistance. Clustered regularly interspaced short palindromic repeats (CRISPR)/CRISPR-associated protein 9 (Cas9) screening is an emerging approach to search for potential essential genes or drug targets using knockout or activation [16]. A CRISPR library consists of thousands of single-guided RNAs (sgRNAs), which target protein-coding genes or noncoding RNAs. Via a knockout approach, essential genes can be identified using negative selection [17]. Another CRISPR/Cas9 strategy developed by Zhang uses a three-unit engineered protein complex, which can activate the transcription of lncRNAs via sgRNAs [18]. With a lncRNA activation library, drug sensitivity genes can be identified by negative selection, while drug resistance genes are abundant in the positive selection results. This strategy was applied to screen a melanoma cell line with vemurafenib as the selection pressure, and the lncRNA EMICERI was identified as a resistance driver. Examples of drug resistance genes identified by CRISPR activation screening include LRP8 [19], CASC11 [20], MYADML2 [21], and PRMT3 [22]. With this potent tool, the molecular mechanisms of sorafenib resistance in HCC or other types of cancers can be easily revealed, as undiscovered targets can also be considered.

Peroxisome proliferator-activated receptor alpha (PPARα), encoded by the PPARA gene, is a nuclear receptor protein that functions as a transcription factor. While expressing in various tissues including the liver, kidneys, hearts, and adipose tissues, its role in the regulation of lipid metabolism and energy homeostasis is crucial [23]. PPARα has been shown to induce a metabolic switch from glycolysis to fatty acid oxidation in cancer [24]. Studies have shown that PPARα activation can induce the gene expressions involved in fatty acid oxidation, and lead to a decrease in glucose uptake and glycolysis. This metabolic switch has been shown to be beneficial for cancer cells, as it provides extra energy for cancer cells to survive in nutrients-deprived environment or hypoxia [25].

In this study, we used a human CRISPR/Cas9 synergistic activation mediator (SAM) pooled library to screen for sorafenib resistance modulators. By negative selection, we identified the lncRNA Linc01056 as sensitive to sorafenib, and knockdown (KD) of Linc01056 resulted in enhanced sorafenib resistance. Our in vivo and in vitro models showed that Linc01056 knockdown could reduce sorafenib sensitivity by metabolic reprogramming. The shift from glycolysis to fatty acid oxidation (FAO) maintains a high level of intracellular ATP, and this change is governed by elevated transcriptional activity of PPARα. Considering the clinical significance identified by human tissue array analysis, we suggest that Linc01056 is a potential drug target for sorafenib-resistant HCC.

## Materials and methods

### Human samples

Human liver cancer tissue microarray chips (LivH180Su08) containing 90 pairs of human HCC samples were obtained from Shanghai OUTDO Biotech Company (China). The associated clinical and pathological information was also provided by the supplier. The collection of human tissues with informed consent was approved by the Medical Institutional Review Boards in Shanghai following the ethical guidelines.

### Cell culture

The MHCC97L cell line with luciferase expression was a gift from Prof. Man Kwan from the Department of Surgery, the University of Hong Kong. The MHCC97L cell line was originated from the parent cell line MHCC97 of an animal model of human HCC LCI-D20 tumour [26]. The PLC/PRF/5 and HepG2 cell line was obtained from the American Type Culture Collection (ATCC; USA). The 293FT cell line (from Invitrogen) was a gift from Prof. Xinyuan Guan from the University of Hong Kong. The cell lines were cultured in high-glucose Dulbecco’s modified Eagle’s medium (Gibco, USA) supplemented with 10% foetal bovine serum (FBS; Gibco) and 1% penicillin/streptomycin (Gibco). For the culture of HEK293FT cells, 1 mM sodium pyruvate (Gibco) was added to the above medium. Cell lines used in this study have been authenticated by STR profiling and were proved to be mycoplasma-free.

### In vitro CRISPR‒Cas9 library screen

The CRISPR‒Cas9 lncRNA activation screen was performed using the human CRISPR 3-plasmid lncRNA SAM pooled library (Addgene #1000000106), which was a gift from Prof Feng Zhang. The library consists of 95,058 sgRNAs targeting 10,504 lncRNAs, with the transcription start site (TSS) of each lncRNA targeted by approximately ten sgRNAs. The sgRNA library was cloned according to the published protocol [18] and amplified using Endura electrocompetent cells following the manufacturer’s instructions. The purified sgRNA library was then packaged into lentiviruses together with the other two components of the SAM system, dCas9-VP64-blast and MS2-P65-HSF1. MHCC97L cells were transduced with dCas9-VP64 and MPHv2 and selected for five days. The stable clones were then subjected to zeocin kill curve analysis before transduction of the sgRNA library at a multiplicity of infection of < 0.3 to ensure that each cell contained a maximum of 1 sgRNA. The cell pool was selected with 300 µg/mL zeocin (InvivoGen, USA). The remaining cells were cultured with a nonlethal dose of sorafenib for 7 days and then maintained until each sgRNA was covered by 500 cells.

Total genomic DNA was isolated using the Zymo Research Quick-gDNA MidiPrep Kit (Zymo Research, USA). PCR amplification was performed on the sgRNA-targeted regions using NEBNext High Fidelity 2x Master Mix (New England Biolabs, USA). Ten pairs of primers with included barcodes were used for PCR. The PCR products were purified by gel electrophoresis prior to gel purification (Qiagen, Germany). The recovered DNA was subjected to next-generation massively parallel amplicon paired-end sequencing (Novogene, China) to determine the presence of sgRNAs.

### Fluorescence in situ hybridization (FISH) and multiplex immunofluorescence staining

FISH was carried out using a staining kit from Servicebio (China) following a modified protocol from the manufacturer. In brief, cells were fixed with 4% paraformaldehyde solution (PFA) and were then permeabilized with 0.5% Triton X-100 at room temperature for 20 min prior to a 10 min digestion with proteinase K at 37 °C. For the paraffin-embedded tissue array, rehydration steps starting with xylene were performed. The tissue sections were then digested with 5 µL/mL proteinase K at 37 °C. The samples were prehybridized at 40 °C for 30 min and were then incubated with the Linc01056 probe at 40 °C overnight. The samples were then incubated with the amplification probe at 40 °C for 45 min. The probes were labelled with the fluorescent dye AF488 at 37 °C for 45 min. Washing steps were performed three times between each staining step, with sequential washing with 2x, 1x, 0.5x and 0.1x saline sodium citrate (SSC) buffer for 10 min each at room temperature. Next, a standard immunofluorescence protocol using an AF568-conjugated secondary antibody was performed, and images were acquired with an LSM 900 confocal microscope (Carl Zeiss, Germany).

Multiplex immunofluorescence staining was performed using the Opal 9-color manual IHC detection kit (Akoya Biosciences, USA) according to the manufacturer’s instruction. Briefly, the FFPE tissue microarray was dewaxed and rehydrate through xylene and graded series of ethanol solutions. After rehydration, the slide was fixed in 10% neutral buffered formalin for 20 min. Antigen retrieval (and antibody removal) was performed in boiling AR6 buffer for 20 min using the microwave. After cooling down to room temperature, the slide is rinse with ddH_2_O and TBST, followed by blocking at room temperature for 10 min. Primary antibodies were incubated for 2 h to overnight, secondary antibodies and Opal fluorophores were incubated for 10 min at room temperature. Rinsing was performed three times with 1x TBST between each incubation step. The cycle of staining steps was repeated for each primary antibody and corresponding Opal fluorophore. Finally, the slide was counterstained by DAPI and then subjected to scanning on the Vectra Polaris platform (Akoya). Image processing and analysis was performed on the InForm® software (Akoya).

### Animal experiments

All animal experiments were approved by the Committee on the Use of Live Animals in Teaching and Research (CULATR) of the University of Hong Kong. The animal experiments were performed in AAALAC-accredited facilities at the Centre for Comparative Medicine Research of the University of Hong Kong.

A total of 1 × 10^6^ luciferase-tagged wild-type or LINC01056-KD MHCC97L cells were subcutaneously injected into the right flanks of NOD.CB17-Prkdc^scid^/J (NOD scid) mice. When the tumours reached 10 mm in diameter, the mice were sacrificed, and the tumours were harvested and cut into small cubes 1 mm in length on each side. One tumour cube was implanted onto the left lobe of the liver of a 5-week-old male BALB/cAnN-nu (Nude) mouse. The growth of orthotopic HCC tumours was monitored by luciferin bioluminescence imaging using an in vivo imaging system (IVIS; PerkinElmer, USA).

### Statistical analysis

Statistical analyses were performed using Prism 9 (GraphPad, USA). Experiments were performed with three replicates unless otherwise stated. Student’s t test was used for two-group comparisons, and one-way ANOVA was used for multigroup comparisons. *A p* value of < 0.05 was considered statistically significant.

## Results

### CRISPRa screening identifies Linc01056 as a candidate regulator of sorafenib sensitivity in HCC

**Fig. 1:**
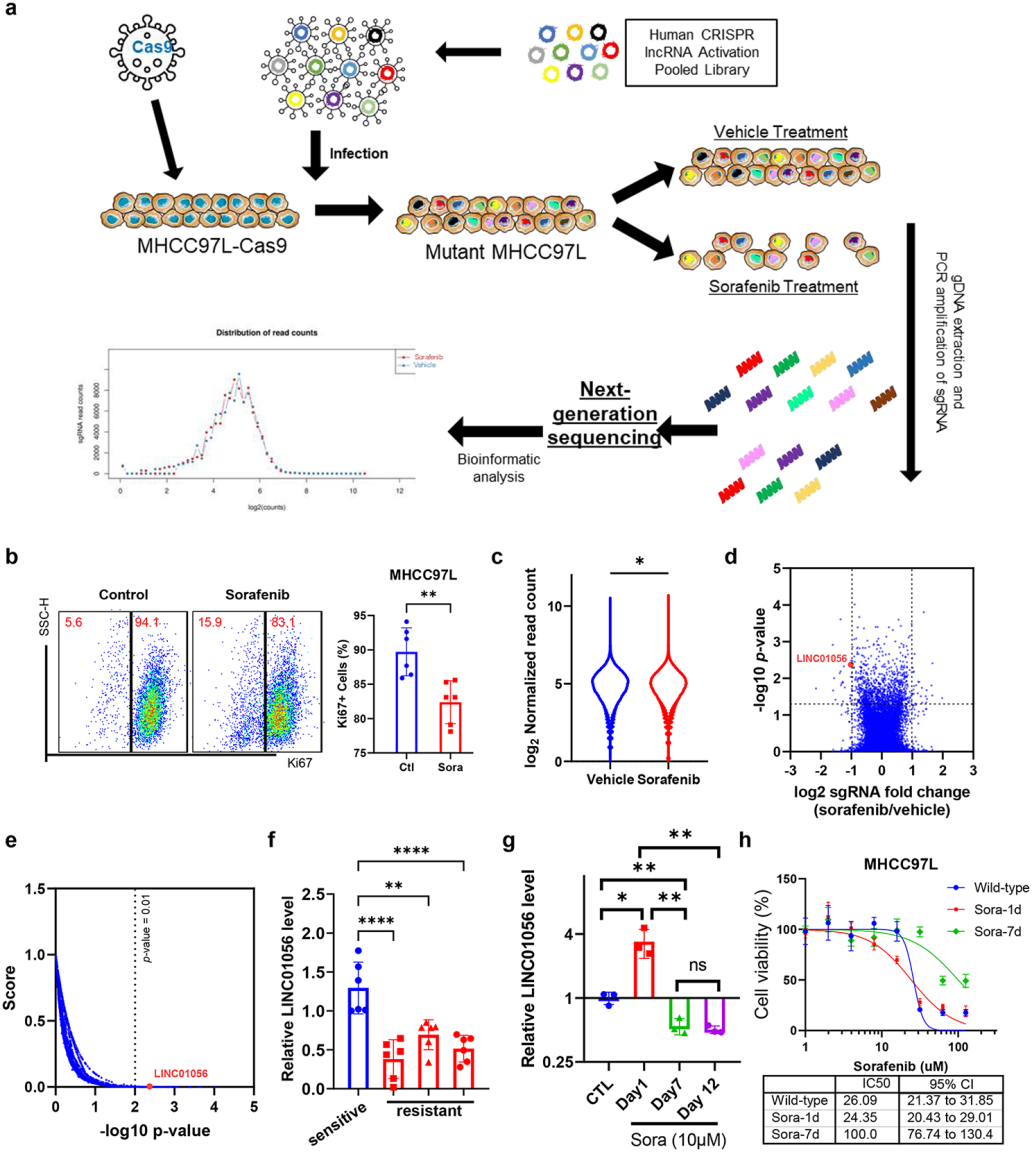
CRISPRa screens identified Linc01056 as a regulator of sorafenib response in HCC cells. (**a**) Flowchart of CRISPRa screening on MHCC97L cells. (**b**) 7-day treatment of sorafenib significantly suppressed the proliferation ability of MHCC97L cells. (**c**) Violin plot of the normalized read count of the sequencing result. The average count of the sorafenib-treated group was slightly higher. (**d**) Volcano plot of the changes of expression of lncRNAs upon 7-day exposure of sorafenib in HCC cells. (**e**) Linc01056 was one of most downregulated lncRNA upon acquisition of sorafenib resistance in HCCs. The acquired sorafenib resistance MHCC97L cells were obtained by prolonged seven-day 5 µM sorafenib treatment. (**f**) In sorafenib-resistant HCC tumour cells, the expression of Linc01056 was potently suppressed. (**g**) HCC cells were exposed to 1-, 7- and 12-day sorafenib at the dose of 10µM, it was observed that 1-day treatment of sorafenib induced Linc01056 expression, while long-term treatment of sorafenib suppressed Linc01056 expression. (**h**) Cell viability of MHCC97L were measured against sorafenib treatment for wild-type or 1-day or 7-day treated cells. *p < 0.05, **p < 0.01, ***p < 0.001

To identify the critical lncRNA regulator of sorafenib sensitivity in HCC, we applied a global screening approach involving a CRISPR/Cas9 lncRNA SAM pooled library containing 96,458 sgRNAs that targeted the TSSs of 10,504 unique lncRNAs [18]. The human HCC cell line MHCC97L, which exhibited a moderate response to sorafenib (Fig. S1a), was used to establish an in vitro model for CRISPRa screening of potential lncRNA regulators. MHCC97L cells with stable expression of the Cas9 protein were transduced with lentiviral sgRNAs and were then treated with vehicle or 5 µM sorafenib for 7 days (Fig. 1a). A 7-day treatment with sorafenib, which provided strong pressure for the selection of positive and negative lncRNA regulators of sorafenib sensitivity in HCC cells, significantly suppressed cell proliferation and induced cell death in MHCC97L cells (Fig. 1b & S1b). MHCC97L cells treated with vehicle or sorafenib were then subjected to next-generation sequencing to identify lncRNAs that were negatively and positively associated with sorafenib sensitivity. Model-based analysis of genome-wide CRISPR/Cas9 activation library was used to identify hits from our CRISPRa screening based on a previous study [27]. Quality control assessment suggested that the sgRNAs resulted in high-purity and clean reads (Fig. S1c). Sorafenib-treated group has a slightly higher average normalised read count, suggesting the successful screening of the lncRNA (Fig. 1c). Using a cut-off of |log_2_FC|≥1, we identified 67 lncRNAs that were downregulated and 79 lncRNAs that were upregulated in the surviving cells after sorafenib treatment (Fig. 1d). The lncRNA-specific sgRNA were confirmed that can effectively activate the corresponding targets when transfected into MHCC97L cells using qPCR (Fig. S1d). From the result of sequencing, Linc01056 was one of the most downregulated lncRNAs in the surviving MHCC97L cells after 7 days of 5µM sorafenib treatment (Fig. 1e). Linc01056 is a lncRNA located at chromosome 20, 63,038,011–63,053,863, with an exon-spiced length of 1,234 nt (Table S1), while lacking protein coding potentials (Fig. S1e). It does not overlap with any known protein-coding genes (Fig. S1f). According to the published dataset GSE30611, Linc01056 mainly presents high copy numbers in breast cancer, gastric cancer, and HCC, while presents low copy numbers in lung cancer, ovarian cancer, and leukaemia [28]. To examine whether Linc01056 expression is suppressed in sorafenib-resistant HCC, we established in vivo-generated sorafenib-resistant HCC tumours according to our previous study [29]. Significant downregulation of Linc01056 expression was observed in sorafenib-resistant HCC tumours compared to their sorafenib-sensitive counterparts (Fig. 1f). In addition, we challenged MHCC97L cells with 10 µM sorafenib for 24 h and 7 days. Intriguingly, we observed an impulsive stimulated expression of Linc01056 in 24 h sorafenib treatment, but significantly suppressed expression after 7-day exposure (Fig. 1g). The sorafenib resistance was greatly enhanced with the reduction of Linc01056 expression after 7-day exposure (Fig. 1h), indicating that the reduced level of Linc01056 in response to sorafenib treatment may be associated with sorafenib resistance. Our previous study illustrated the change in transcription factor profile in sorafenib-treated MHCC cells [29], where ETS Proto-Oncogene 1 (ETS1) was found to be a responder of sorafenib. To check if ETS1 is also responsible for the transcription of Linc01056, we predicted the binding motif of ETS1 in JASPER and located that AGGAAG from -895 to -900 before the promoter of Linc01056 is a key binding motif (Fig. S1g). Chromatin-immunoprecipitation (ChIP) assay proved the binding of ETS1 to the promoter region of Linc01056 with increased level after sorafenib treatment (Fig. S1h) Induction of Linc01056 expression was suppressed with siETS1 (Fig. S1i), and the sorafenib sensitivity was also reduced (Fig. S1j). The results suggested that ETS1 is a transcriptional regulator of Linc01056. Collectively, we suggest that expression of Linc01056 has a role in response to sorafenib of HCC cells.

### Linc01056 is essential for the sorafenib sensitivity of HCC in vitro and in vivo.

**Fig. 2:**
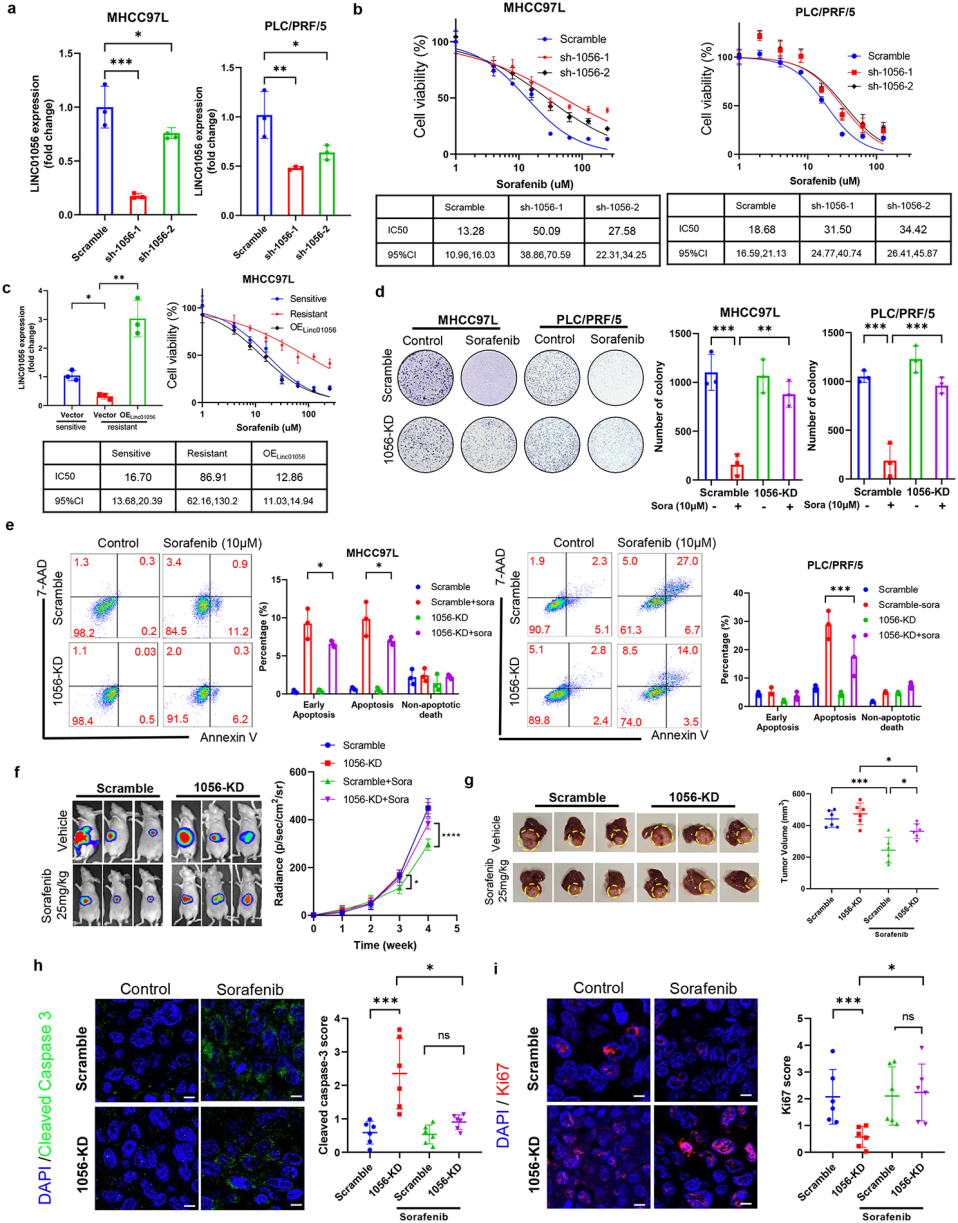
Linc01056 was required for sorafenib response in HCC. (**a**) Knockdown of Linc01056 in MHCC97L and PLC/PRF/5 cells by shRNA interference. (**b**) Knockdown of Linc01056 increased cell viability in sorafenib-treated HCC cells. (**c**) Rescue of Linc01056 potentiated the resistant HCC cells to sorafenib treatment. (**d**) Knockdown of Linc01056 improved colonic formation of HCC cells in the presence of sorafenib. (**e**) Knockdown of Linc01056 reduced sorafenib-induced apoptosis in HCC cells. Stable knockdown of Linc01056 (**f**) accelerated in vivo tumour growth and (**g**) end-point tumour size, (**h**) reduced expression of cell apoptosis marker cleaved caspase-3 and (**i**) promoted the cell proliferation marker Ki67. *p < 0.05, **p < 0.01, ***p < 0.001

To identify the functional role of Linc01056 in the sorafenib sensitivity of HCC cells, we first generated MHCC97L and PLC/PRF/5 cells with stable knockdown of Linc01056. Significant suppression of Linc01056 was observed in HCC cells stably expressing the shRNA plasmids targeting Linc01056 (Fig. 2a). With a better knockdown effect, sh-1056-1 was chosen for the remaining study. Cell viability assays revealed that knockdown of Linc01056 in HCC cells resulted in a significantly attenuated response to sorafenib treatment (Fig. 2b). To confirm there is no off-target effect, we rescued expression of Linc01056 in resistant MHCC97L cells established by 7-day sorafenib intervention by a Linc01056-expressing plasmid. Rescue of Linc01056 potentiated the resistant HCC cells to sorafenib treatment (Fig. 2c). While knockdown of Linc01056 showed a minimal effect on cell growth in the absence of sorafenib, cells with lower Linc01056 expression exhibited a stronger colony formation ability upon long-term sorafenib treatment (Fig. 2d). Linc01056 knockdown significantly reduced apoptosis in HCC cells upon sorafenib treatment compared to that in vector control cells (Fig. 2e). Moreover, treatment of HCC cells with a nontoxic concentration of sorafenib potently suppressed the in vitro motility as well as invasion through the extracellular matrix, and these abilities were significantly restored upon knockout of Linc01056 expression (Fig. S2a & S2b). To investigate if the level of Linc01056 is related to the sorafenib resistance, we performed a CRISPR-KO targeting Linc01056 also on the MHCC97L and PLC/PRF/5 cell line (Fig. S2c). The CRISPR-KO cells showed a higher IC50 value to sorafenib than Linc01056-knockdown cells (Fig. 2b, Fig.S2d), and the results of the apoptosis assay was consistent (Fig. S2e).

To examine the role of Linc01056 in HCC in vivo, we established an orthotopic HCC model in mice via implantation of luciferase reporter-expressing MHCC97L cells with or without Linc01056 knockdown. We observed that Linc01056 knockdown significantly reduced the in vivo tumour response to sorafenib treatment without a change in body weight (Fig. S2f), as indicated by the rate of tumour growth (Fig. 2f). At the end of the study, the livers were harvested, and Linc01056 knockdown was found to result in a larger tumour size and higher tumour weight in sorafenib-treated mice bearing HCC tumours (Fig. 2g). Increased expression of Ki67 and a decreased level of cleaved caspase-3 were observed in tumour tissues from sorafenib-treated mice implanted with Linc01056-knockdown MHCC97L cells compared to those in mice implanted with the corresponding vector control cells (Fig. 2h and 2i). Furthermore, Linc01056 knockdown greatly increased the probability of lung metastasis of MHCC97L cells in mice exposed to sorafenib (Fig. S2g). Taken together, our findings suggest that Linc01056 expression is required for the response of HCC cells to sorafenib treatment both in vitro and in vivo.

### Loss of Linc01056 mediates the metabolic switch towards FAO in sorafenib-treated HCC

**Fig. 3:**
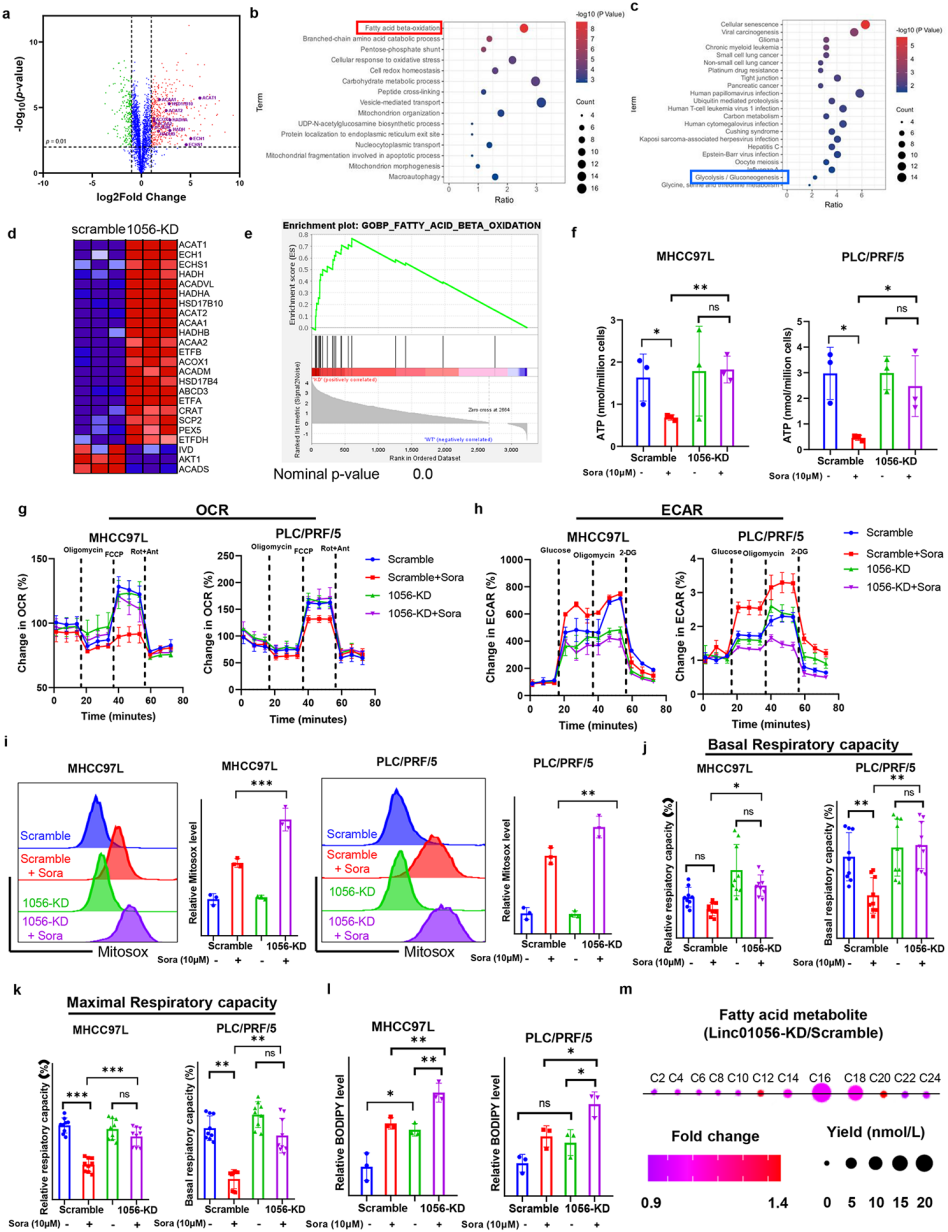
Linc01056 knockdown induced metabolic shift towards fatty acid oxidation. (**a**). Proteomic analysis on sorafenib-treated MHCC97L cells with or without Linc01056 knockdown. Pathway enrichment on differential gene expression showed that (**b**) increased genes enriched in pathways related to fatty acid oxidation, while **c)** reduced genes enriched in pathways related to glycolysis/gluconeogenesis. (**d**) Changes in expression of FAO-related proteins upon Linc01056 knockdown. (**e**) GSEA analysis showed enrichment of genes related to FAO. (**f**) Linc01056 knockdown maintained cellular ATP level upon sorafenib treatment in HCC cells. Knockdown of Linc01056 (**g**) increased the OCR and (**h**) decreased the ECAR in HCC cells in the presence of sorafenib. Linc01056 knockdown (**i**) increased mitochondrial ROS, and (**j**) maintained the basal respiratory and (**k**) maximal respiratory capacity in sorafenib-treated HCC cells. (**l**) Linc01056 increased fatty acid storage in sorafenib-treated HCC cells.(**m**) Linc01056 knockdown increased content of C16 intermediates of fatty acid in sorafenib-treated HCC cells. *p < 0.05, **p < 0.01, ***p < 0.001

To further explore the possible mechanisms underlying the reduced sorafenib response in HCC cells with Linc01056 knockdown, we performed proteomics analysis to compare the protein expression profile between sorafenib-treated MHCC97L cells transduced with the vector control plasmid or the Linc01056 shRNA plasmid. Differential changes in protein expression were observed (Fig. 3a). We then shortlisted the upregulated and downregulated proteins upon Linc01056 knockdown (Fig. 3b and 3c) and performed Gene Ontology (GO) analysis to determine the possibly enriched biological processes. We found that the proteins upregulated by Linc01056 knockdown were enriched primarily in FAO-related terms, while the downregulated proteins were enriched in glycolysis-related terms, indicating that Linc01056 knockdown may activate a metabolic switch from glycolysis towards FAO upon sorafenib pressure (Fig. 3d). Consistent enriched pathways were obtained from the proteomics analysis of sorafenib-treated PLC/PRC/5 cells (Fig. S3a). Gene set enrichment analysis (GSEA) confirmed that MHCC97L cells with Linc01056 knockdown showed higher enrichment of FAO activity (Fig. 3e). The expression of genes related to FAO was significantly increased but that of glycolysis-associated genes was markedly reduced in sorafenib-treated HCC cells with Linc01056 knockdown (Fig. S3b & S3c).

The acquisition of sorafenib resistance in HCC cells requires a high level of intracellular energy to maintain cell growth and survival under sorafenib pressure [30]. We found that Linc01056 knockdown in MHCC97L and PLC/PRF/5 HCC cells resulted in higher levels of intracellular ATP, indicating that the metabolic switch from glycolysis towards FAO may confer an advantage on energy production in HCC cells (Fig. 3f). We then profiled the metabolic characteristics of sorafenib-treated HCC cells with Linc01056 knockdown using Seahorse XF assays. Knockdown of Linc01056 in HCC cells significantly increased the oxygen consumption rate (OCR) and decreased the extracellular acidification rate (ECAR), confirming that HCC cells with Linc01056 knockdown preferentially utilize oxidative phosphorylation (OXPHOS) instead of glycolysis to generate ATP (Fig. 3g and h). In addition, the mitochondrial reactive oxygen species (mtROS) level was markedly elevated upon Linc01056 knockdown in HCC cells (Fig. 3i). Knockdown of Linc01056 significantly increased the basal and maximal respiratory capacities of sorafenib-treated HCC cells (Fig. 3j and k). Knockdown of Linc01056 in HCC cells resulted in reduced glucose uptake and suppressed extracellular lactic acid production, as well as cellular LDH activity (Fig. S3d–f), but significantly increased the consumption of intracellular free fatty acids (Fig. 3l). Metabolic profiling of fatty acids derived from sorafenib-treated MHCC97L cells with Linc01056 knockdown suggested an increased content of C16 intermediates compared with that in the vector control counterpart cells (Fig. 3m). Collectively, these observations suggested that Linc01056 knockdown resulted in a metabolic switch from glycolysis towards FAO in sorafenib-treated HCC cells that increased the efficiency of energy production.

### Linc01056 loss-induced FAO is required for the acquisition of sorafenib resistance in HCC

**Fig. 4:**
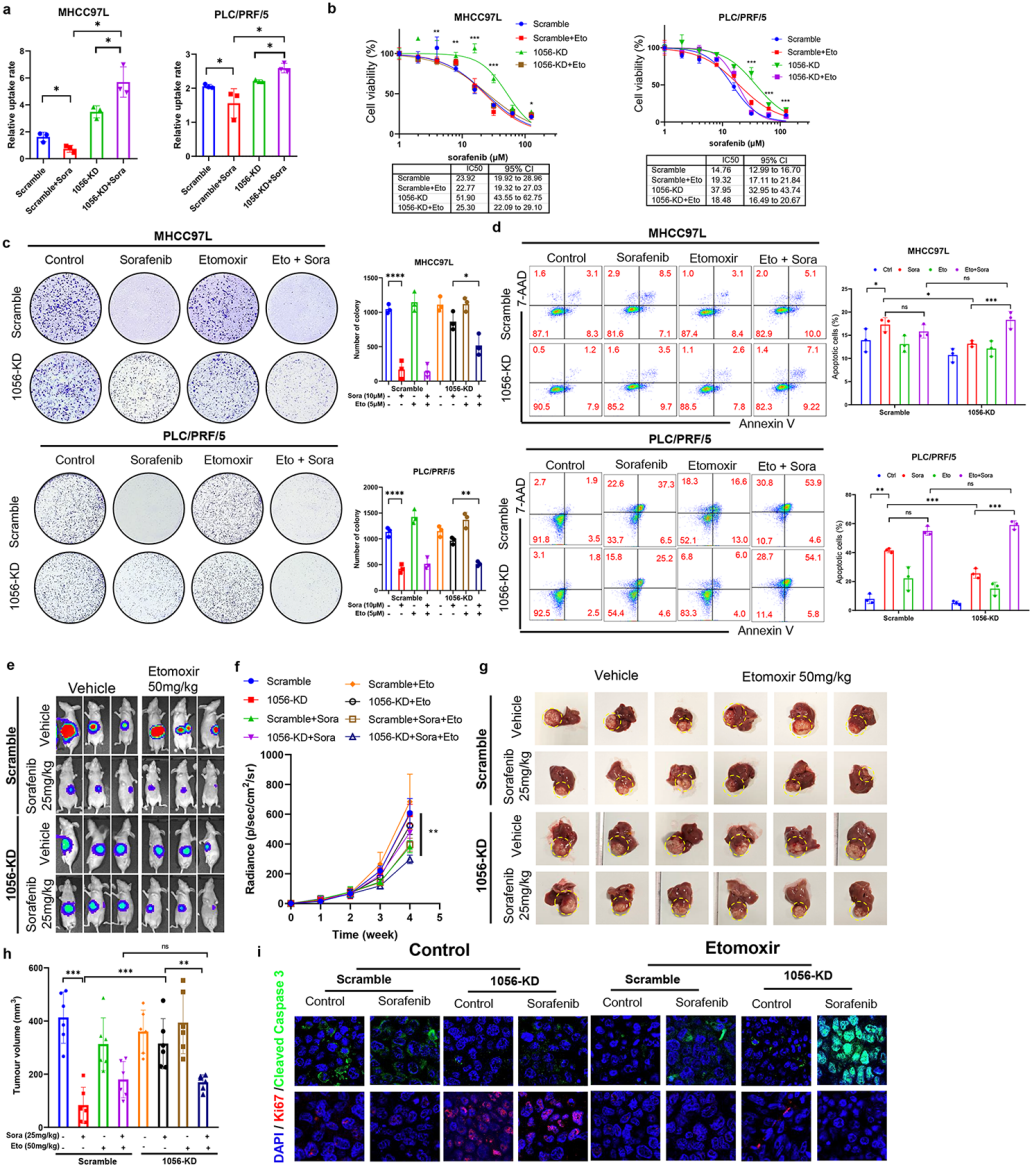
FAO inhibition sensitised Linc01056-knockdowned HCC cells upon sorafenib treatment. (**a**) Knockdown of Linc01056 resulted in an increase in fatty acid uptake in HCC cells, which was further augmented under sorafenib treatment. FAO suppression by etomoxir (**b**) increased cytotoxicity of sorafenib, (**c**) suppressed colonic capacity, and (**d**) induced apoptosis in HCC cells with Linc01056 knockdown. FAO suppression by etomoxir (**e & f**) reduced in vivo tumour growth and (**g & h**) end-point tumour size, (**i**) increased expression of cell apoptosis marker cleaved caspase-3 and reduced the cell proliferation marker Ki67 in sorafenib-treated in vivo HCC tumours without Linc01056 knockdown. *p < 0.05, **p < 0.01, ***p < 0.001

Both glycolysis and FAO have been reported to be hyperactive in HCC cells [31, 32]. To explore whether the Linc01056 knockdown-induced metabolic reprogramming from glycolysis towards FAO is due to direct inhibition of glycolysis by Linc01056, we cotreated HCC cells with the glycolytic inhibitor 2-DG in the presence of sorafenib. Inhibition of glycolysis by 2-DG sensitized HCC cells to sorafenib (Fig. S4a). Consistent with this finding, apoptosis induction by sorafenib treatment in HCC cells was increased in the presence of 2-DG regardless of Linc01056 knockdown surprisingly (Fig. S4b). These results suggested that alone, inhibition of glycolysis in sorafenib-treated HCC cells was not sufficient to induce sorafenib resistance. Hence, we hypothesized that Linc01056 knockdown confers sorafenib resistance through direct activation of FAO in sorafenib-treated HCC cells. Increased fatty acid uptake was observed in HCC cells with Linc01056 knockdown (Fig. 4a), as was enhanced expression of genes related to fatty acid uptake (Fig. S4c). Interestingly, we did not observe significant changes in the expression of genes associated with de novo lipogenesis (Fig. S4d). These observations suggested that the Linc01056 knockdown-associated metabolic switch was directly related to the activation of fatty acid β-oxidation.

To test whether FAO activation induced by Linc01056-KD contributes to sorafenib resistance in HCC cells, we applied etomoxir, a CPT1 inhibitor that blocks fatty acid transport and utilization in mitochondria, to suppress FAO. Etomoxir showed a minimal effect on sorafenib sensitivity in HCC cells transduced with vector but significantly improved the HCC cell response to sorafenib in HCC cells with Linc01056 knockdown (Fig. 4b). Similarly, etomoxir strongly reduced the colony formation capacity of HCC cells with Linc01056 knockdown under sorafenib treatment (Fig. 4c). Treatment of etomoxir re-sensitized the Linc01056 knockdown HCC cells by increasing the sorafenib-induced apoptosis (Fig. 4d). Moreover, etomoxir further increased the inhibitory effect of sorafenib on the in vitro motility and invasion of HCC cells with Linc01056 knockdown (Fig. S5a & S5b). In vivo, etomoxir was applied to investigate the role of FAO activation in Linc01056 knockdown-induced sorafenib resistance in the orthotopic HCC mouse model. Etomoxir treatment improved the sorafenib response of orthotopic HCC tumours by suppressing their growth (Fig. 4e, f) and resulted in smaller tumour sizes (Fig. 4g, h). The improvement in the sorafenib response induced by etomoxir in HCC tumours with Linc01056 knockdown was further supported by the reduced expression of Ki67 and increased level of cleaved caspase-3 in the tumour tissues (Fig. 4i). Furthermore, etomoxir further enhanced the inhibitory effect of sorafenib on the lung metastasis of MHCC97L cells with Linc01056 knockdown (Fig. S5c). These observations confirmed that FAO activation played an essential role in mediating sorafenib resistance in HCC cells upon Linc01056 knockdown.

### Linc01056 loss activates PPARα-mediated transcription of FAO-associated genes

**Fig. 5:**
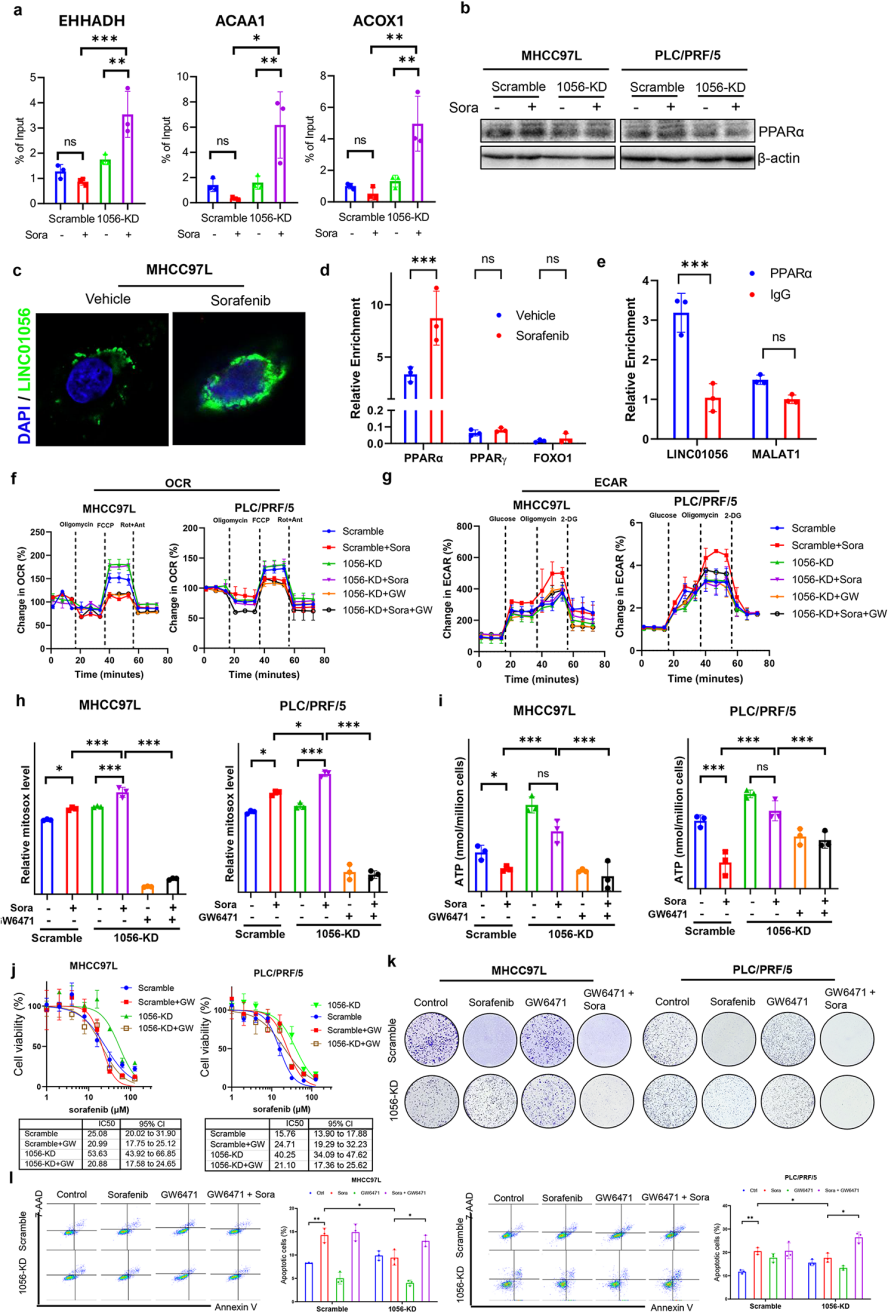
Linc01056 interfered PPARα transcription activity-associated FAO activation. (**a**) Linc01056 knockdown induced binding of PPARα on to the promoter region of FAO-related genes. (**b**) Linc01056 knockdown did not change the protein expression of PPARα in HCC cells. (**c**) Linc01056 located in the cytoplasm of MHCC97L cells with or without sorafenib treatment. (**d**) Linc01056 specifically bound to PPARα but not PPARγ or FOXO1. (**e**) PPARα specifically bound to Linc01056 but not other lncRNA like MALAT1. Suppression of PPARα activity by GW6471 (**f**) reversed the increase of OCR and (**g**) decrease of ECAR in Linc01056-knockdown HCC cells upon sorafenib treatment. GW6471 inhibited (**h**) production of mitochondrial ROS, (**i**) intracellular ATP, (**j**) cell viability, (**k**) colonic capacity, and (**l**) apoptosis of Linc01056-knockdown HCC cells upon sorafenib treatment. *p < 0.05, **p < 0.01, ***p < 0.001

Given that Linc01056 functions in regulating sorafenib sensitivity by maintaining intracellular energy metabolism homoeostasis, we hypothesized that Linc01056 critically regulates PPARα, the cellular sensor that suppresses glycolysis, while inducing FAO activation [33]. Indeed, knockdown of Linc01056 in HCC cells resulted in transcriptional activation of PPARα-specific target genes, including EHHADH, ACAA1 and ACOX1 (Fig. S6a) and resulted in nuclear localization of PPARα (Fig. S6b), confirming the transcriptional activation of PPARα. The ChIP results suggested that PPARα bound to the transcriptional activation binding site in the promoter regions of FAO-related genes (Fig. 5a) upon Linc01056 knockdown, a finding that confirmed the regulatory role of Linc01056 in PPARα transcriptional activity. LncRNAs regulate the activity of transcription factors via multiple mechanisms [34]. Interestingly, we did not observe obvious changes in the mRNA and protein expression of PPARα (Fig. S6c & Fig. 5b), suggesting that Linc01056 regulates PPARα transcriptional activity through posttranslational mechanisms. In situ hybridization revealed that Linc01056 localized to the cytoplasm but not the nucleus in MHCC97L cells regardless of sorafenib treatment (Fig. 5c). In addition, the RIP assay results showed that cytoplasmic Linc01056 could specifically bind to PPARα but not PPARɤ or FOXO1 in MHCC97L cells (Fig. 5d). Moreover, the immunoprecipitation assay results confirmed that PPARα bound to Linc01056 but not another lncRNA, MALAT1 (Fig. 5e). These observations indicated that Linc01056 specifically bound to PPARα to prevent its nuclear localization and transcriptional activity.

To explore the role of PPARα in mediating FAO activation upon Linc01056 knockdown, we treated HCC cells with the PPARα-specific inhibitor GW6471. Treatment with GW6471 significantly reversed the Linc01056 knockdown-induced increase in the OCR in sorafenib-treated HCC cells, while the increase of ECAR upon PPARα inhibition was subtle (Fig. 5f and g). Moreover, GW6471 potently decreased the basal and maximal respiratory capacities of sorafenib-treated HCC cells with Linc01056 knockdown (Fig. S6d & S6e). Thus, the induction of mtROS production upon Linc01056 knockdown was strongly inhibited by GW6471 (Fig. 5h), and intracellular ATP production was partially reduced upon GW6471 treatment (Fig. 5i). These observations confirmed the role of PPARα in mediating FAO activation upon Linc01056 knockdown in sorafenib-treated HCC cells. Furthermore, inhibition of PPARα by GW6471 in HCC cells with Linc01056 knockdown restored sorafenib sensitivity, as measured by a cell viability assay (Fig. 5j), and suppressed colony formation (Fig. 5k). Sorafenib-induced apoptosis was further increased in HCC cells with Linc01056 knockdown by GW6471 treatment (Fig. 5l). In addition, GW6471 treatment further increased the inhibitory effect of sorafenib on the in vitro motility and invasion of HCC cells with Linc01056 knockdown (Fig. S6f & S6g). Collectively, these observations suggested that activation of PPARα-associated gene transcription mediates FAO activation in sorafenib-treated HCC cells with Linc01056 knockdown.

### Clinicopathological significance of Linc01056 in HCC

**Fig. 6:**
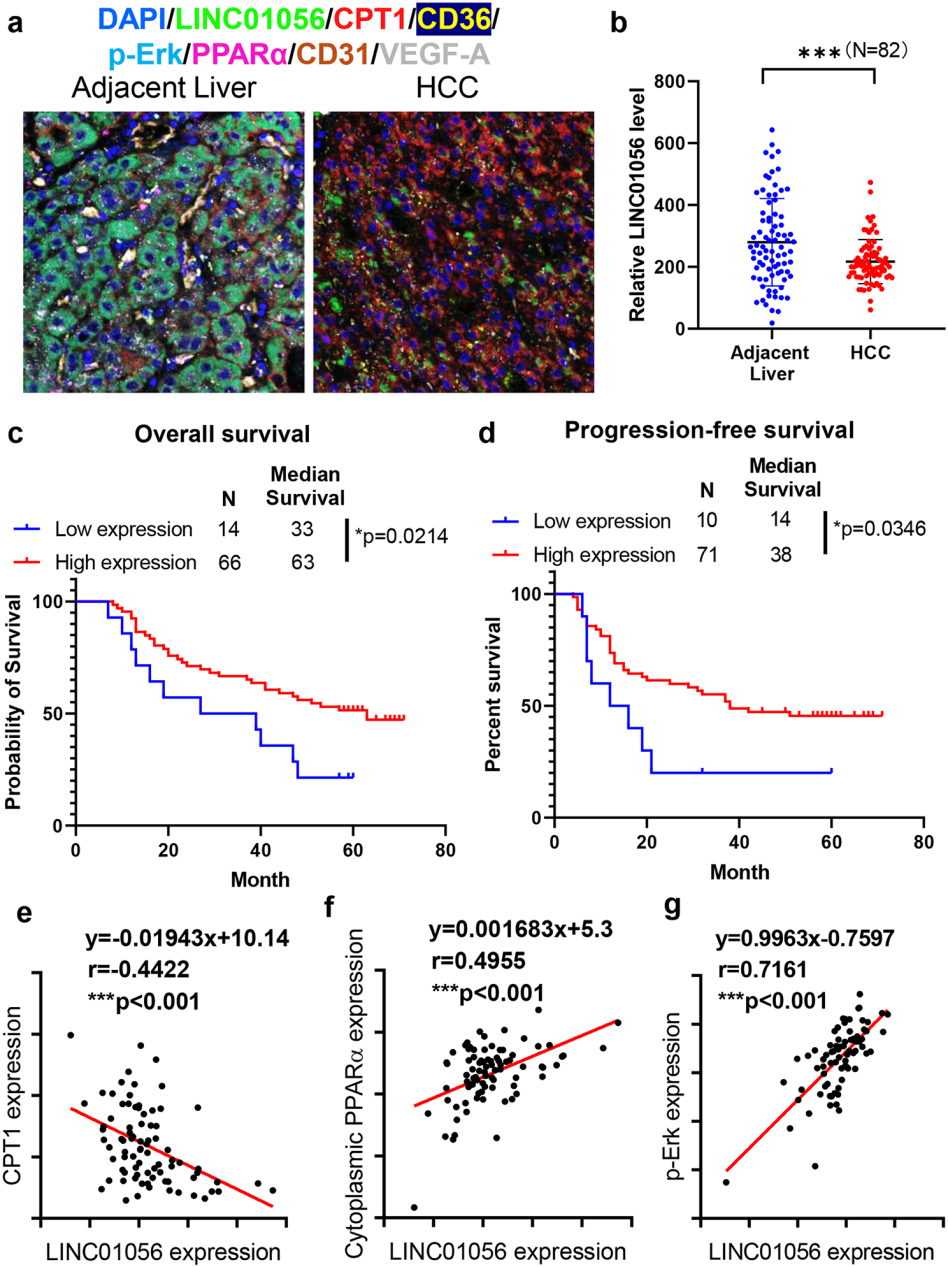
Clinicopathological significance of Linc01056 in HCC. (**a**) representative image of multiplex staining on tissue microarray of human HCC samples. (**b**) Expression of Linc01056 was significantly downregulated in HCC compared to adjacent liver. High expression of Linc01056 predicted the good prognosis of (**c**) overall survival and (**d**) progression-free survival of HCC patients. (**e**) Expression of Linc01056 was negatively correlated with FAO marker CPT1 in human HCC samples. (**f**) Expression of Linc01056 was positively correlated with the cytoplasmic localization of PPARα. (**g**) Expression of Linc01056 was positively correlated with the sorafenib sensitivity marker p-Erk. *p < 0.05, ***p < 0.001

To determine the clinicopathological significance of Linc01056 in HCC, we examined the expression of Linc01056 and Linc01056-related signalling molecules that we identified in this study using a combination of in situ hybridization and multiplex immunofluorescence. The tissue array containing tumour sections from 90 patients was analysed (Fig. S7a; representative image in Fig. 6a, patient information in Supplementary Table S3). We found that the cytoplasmic expression of Linc01056 was significantly downregulated in HCC tumour tissue compared to non-tumour adjacent tissue (Fig. 6b), consistent with the data reported from two other HCC patient cohorts, GSE62232 and GSE76297 (Fig. S7b & S7c). The expression of Linc01056 in HCC tissues and overall survival or progression-free survival in the patients are negatively correlated (Fig. 6c and 6d). Also, the level of Linc01056 is significantly lower in patients with recurrence (Fig. S7d). However, Linc01056 expression was not associated with HCC stage (Fig. S7e) or tumour size (Fig. S7f). To identify the clinical correlation between Linc01056 expression and the expression of the signalling molecules identified in this study, we performed staining for PPARα and the fatty acid transporter CD36 and CPT1, as representatives of FAO activity, in HCC tumours and quantified their expression levels. We found significant negative correlations between Linc01056 expression and the expression of CPT1 (Fig. 6e). The expression of Linc01056 was positively correlated with cytoplasmic localisation of PPARα (Fig. 6f). Notably, PPARα was expressed significantly higher in the sorafenib non-responder group compared to the responder group, as observed in the published patient cohort GSE109211 (Fig. S7g) Consistently, expression of PPARα in HCC was positively correlated with FAO-related CPT1 and CD36 expression from our immunostaining (Fig. S7h & S7i). Phospho-Erk was previously reported as a predictive marker of the sorafenib response in HCC patients, and we observed that Linc01056 expression was positively correlated with the phospho-Erk level in HCC tissues [35], indicating the clinical association of Linc01056 expression with the sorafenib response in HCC patients (Fig. 6g). Collectively, our findings indicate the clinicopathological significance of Linc01056 expression in HCC.

## Discussion

In this study, we found that HCC cells with Linc01056 knockdown were more resistant to sorafenib treatment, a characteristic associated with activation of FAO in these cells. The role of FAO in HCC has been extensively reported. A large-scale gene expression data analysis of 7 cohorts showed that the HCC subtype with preferential FAO was associated with a poor clinical prognosis in HCC patients [36]. In addition, a subgroup of HCC cells expressing activated β-catenin was found to be addicted to fatty acids, and these cells exhibited lower glycolytic activity but intense FAO activity, which promoted HCC development [37]. Mechanistically, FAO was activated during metabolic stress, which facilitated cell survival and therefore accelerated tumour progression. Increased FAO was suggestive of poor overall survival and disease recurrence post-surgery [38]. It was found that different types of cells may undergo reprogramming to FAO to support HCC progression. Our previous study revealed that HCC cells undergo reprogramming to accelerate FAO, which facilitates the production of intracellular ATP and therefore promotes HCC progression [39]. Other studies revealed that FAO activation in tumour-associated macrophages led to increased inflammasome-associated cytokine release that promoted HCC tumorigenesis, progression and metastasis [40, 41]. Chen et al. found that a small population of tumour-initiating stem-like cells in HCC tumours underwent a switch to preferential use of FAO via metabolic reprogramming, which facilitated their self-renewal ability [42]. Here, we observed that in the context of sorafenib resistance, HCC tumour cells preferentially used FAO instead of glycolysis to increase energy production, which facilitate their adaptation to stresses induced by sorafenib challenge and therefore led to drug resistance. This observation echoed the finding in another study showing that metabolic reprogramming from glycolysis to FAO conferred platinum resistance on cancer cells [25]. Therefore, it has been proposed that FAO activation may serve as a metabolic checkpoint that indicates tumour progression and a poor clinical prognosis in HCC patients [43]. However, the mechanism by which FAO contributes to sorafenib resistance in HCC is incompletely understood. Apart from providing more energy, there are also reports that FAO might protect cancer cells from chemotherapy-induced apoptosis by increasing the lipid synthesis on the mitochondrial membrane [44], or maintaining the cancer cell stemness via the CD96-Src-Stat3 signalling pathway [45]. In our present study, we found that knockdown of Linc01056 in HCC cells led to a higher level of intracellular ATP, which could be a metabolic advantage that increases the ability of tumour cells to overcome the stress caused by sorafenib challenge.

We found that PPARα plays an important role in mediating sorafenib resistance by regulating and restoring the balance between FAO and glycolysis. Transcriptional activation of PPARα upon loss of Linc01056 induced the expression of FAO-associated genes while inhibiting the expression of glycolysis-associated genes, therefore reprogramming energy metabolism in sorafenib-treated HCC cells. PPARα is a ligand-activated transcriptional factor that activates its target gene by binding to the peroxisome proliferator response element (PPRE) in the promoter region. PPARα consists of four functional modules, including a DNA binding domain and a ligand binding domain. The DNA binding domain utilizes two zinc finger proteins to look for the PPRE. Upon activation, PPARα dimerizes with RXRα to undergo conformational changes and promote the downstream transcription of the target genes. PPARα activation promotes the uptake, utilization, and catabolism of fatty acids by upregulating the expression of genes involved in fatty acid transport, binding, and activation as well as the enzymes involved in mitochondrial and peroxisomal FAO [46–50]. These changes result in an increased rate of FAO, which leads to a decrease in the availability of fatty acids for other metabolic pathways, such as triglyceride synthesis. In addition to promoting FAO, PPARα activation also influences the balance between FAO and glycolysis by regulating the expression of genes involved in glucose metabolism. For example, PPARα activation can lead to downregulation of genes encoding glycolytic enzymes, such as phosphofructokinase [51, 52], and upregulation of genes encoding gluconeogenic enzymes, such as phosphoenolpyruvate carboxykinase [53, 54]. These changes result in a decrease in glycolysis and an increase in gluconeogenesis, which further shifts the balance towards FAO. Previous studies have shown that lncRNA can bind onto transcription factors or proteins to regulate gene expression by functional changes or affecting its nuclear translocation [55–57]. From our results of the RIP assay and IP assay, we observed that Linc01056 and PPARα bind to each other. With the FISH, we observed that PPARα was present mostly in the cytoplasm when Linc01056 was expressed normally, and while Linc01056 was KD, PPARα had showed an increase in nuclear translocation, and further increased by sorafenib treatment. We suggested that the binding prevent its association with the promotor regions of its target genes, indicating that the regulatory effect of Linc01056 on fatty acid metabolism is associated with the transcriptional activity of PPARα. However, this finding did not rule out the possibility that PPARα is involved in regulating the balance of FAO and glycolysis in HCC cells via an indirect mechanism. PPARα activation can also regulate the balance between FAO and glycolysis through indirect mechanisms. For example, PPARα can form heterodimers with other nuclear receptors, such as retinoid X receptor (RXR) [58], and interact with coregulators, such as PPARγ coactivator 1-alpha (PGC-1α) [59], to modulate the transcription of genes involved in FAO and glycolysis. In addition, the increased rate of FAO leads to an increase in the cellular level of citrate, an allosteric inhibitor of phosphofructokinase [51]. This inhibition results in a decreased rate of glycolysis, further promoting FAO. The exact role of PPARα in mediating Linc01056-associated FAO induction needs further investigation.

We observed that Linc01056 regulates the transcriptional activity of PPARα, therefore altering the expression of PPARα-targeted genes. LncRNAs may regulate gene transcription via multiple mechanisms. The transcribed Linc01056 sequence was not located at a neighbouring loci of PPARα or any of its downstream genes, suggesting that the changes in gene expression were not related to impairment of gene expression by a physical association of Linc01056 transcripts with related chromatin loci [60]. Moreover, the mRNA and protein levels of PPARα were not significantly altered upon knockdown of Linc01056 in HCC cells, suggesting that Linc01056 does not directly regulate the transcription or the protein stability of PPARα and thus regulates the transcriptional activity of PPARα as an epigenetic regulator at the posttranscriptional level [34]. Previous studies have revealed that lncRNAs may interact with the transcriptional machinery and, as a result, activate or suppress downstream gene expression. For instance, lncRNA GAS5 can directly bind to the WW domain of YAP protein to promote the nuclear export of endogenous YAP and facilitate the degradation of the target [57]. LncTCF7 can recruit the SWI/SNF complex to the TCF7 promoter as a guide molecule to trigger TCF7 transcription [61]. The lncRNA HOTAIR can interact with the histone methylation modification complex on several target genes and therefore induce relocalisation of the PRC2 complex, resulting in changes in the histone methylation pattern to alter gene transcription [62, 63]. The lncRNA XIST may interact with the transcription factor EZH2 and therefore suppress the transcription of its target gene KLF2 [64]. Here, using RIP, we found that Linc01056 has a high affinity for PPARα compared to other lncRNAs, such as MALAT1, under exposure to sorafenib in HCC cells, suggesting that Linc01056 can specifically interact with the PPARα protein and therefore regulate its transcriptional activity. This conclusion is consistent with the conclusion from a previous study determining that a lncRNA has selectivity for binding to a transcription factor protein as a decoy molecule to impair the function of the bound transcription factor, thus preventing downstream gene expression [65, 66].

In conclusion, in this study, we applied a CRISPR/Cas9 screening approach to identify the critical lncRNA, Linc01056, driving sorafenib resistance in HCC. Linc01056 was significantly upregulated upon short-term sorafenib challenge but was repressed in HCC cells with acquired sorafenib resistance in vitro and in vivo in tumours derived from these cells. Knockdown of Linc01056 attenuated the sensitivity of HCC cells to sorafenib treatment, thus resulting in sorafenib resistance in HCC tumours in vivo. Knockdown of Linc01056 in HCC cells increased fatty acid consumption and suppressed glycolysis, leading to a metabolic switch that favoured higher intracellular energy production. Inhibition of FAO restored sorafenib sensitivity in HCC cells with Linc01056 knockdown. Mechanistically, PPARα was activated upon Linc01056 knockdown, which in turn induced the transcription of FAO-associated genes while repressing glycolysis-associated genes. Inhibiting PPARα activation in the context of Linc01056 knockdown restored sorafenib sensitivity in HCC cells. Linc01056 acted as a decoy for PPARα in HCC cells to block its transcriptional activity. Clinically, the expression of Linc01056 was correlated with optimal overall and progression-free survival outcomes in HCC patients and was associated with the sorafenib response, as determined by experiments using phospho-Erk as a predictive marker. High expression of Linc01056 indicated low FAO activity in HCC tissues. Our study elucidated an important epigenetic regulator and potential target in the regulation of the sorafenib response in HCC.

### Electronic supplementary material

Below is the link to the electronic supplementary material.

Supplementary Material 1

## Data Availability

No datasets were generated or analysed during the current study.
