# Supplementary material for: Loss of lncRNA LINC01056 leads to sorafenib resistance in HCC
Source: Mol Cancer. 2024 Apr 6;23:74. doi: 10.1186/s12943-024-01988-y (PMC10998324; doi:10.1186/s12943-024-01988-y)
Supplement: Supplementary file 1 — Supplementary Material 1 [file 12943_2024_1988_MOESM1_ESM.docx]

**Supplementary Materials**

**Supplementary Figures**

**
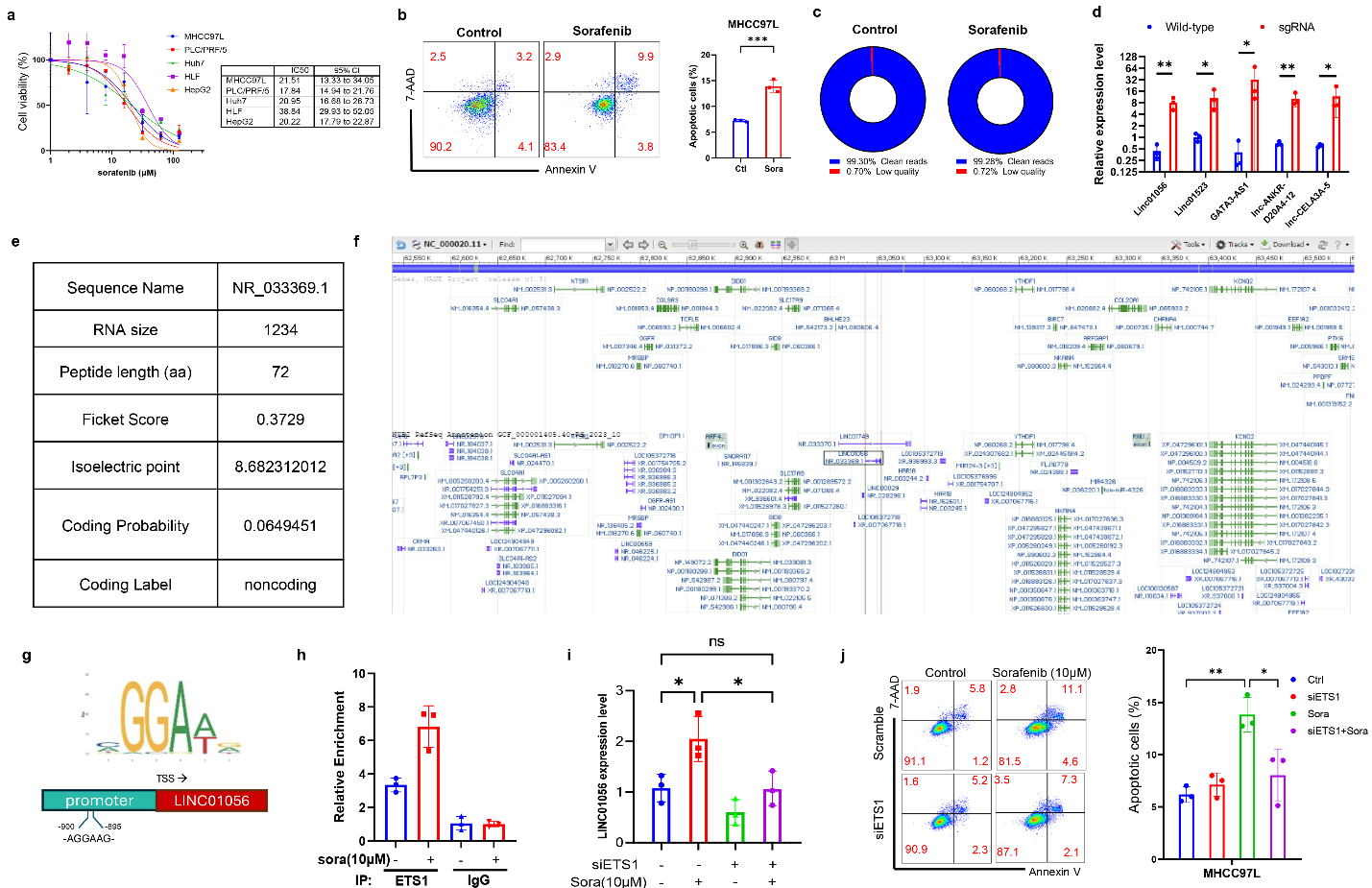
**

**Figure S1 CRISPRa screen of lncRNAs responding to sorafenib treatment in HCC cells. a.** Cytotoxicity of sorafenib to different HCC cell lines at 24 h. **b.** 7-day treatment of sorafenib induced potent apoptosis in MHCC97L cells. **c.** Treatment of sorafenib did not interfere the overall quality of CRISPRa library. **d.** SgRNAs activated the corresponding target lncRNA when transfected into MHCC97L cells. **e.** Linc01056 showed no protein-coding potentials. **f.** Linc01056 does not overlap with other known protein-coding genes. **g.** ETS1 is predicted to be the transcription factor of LINC01056. **h.** ChIP assay confirmed the binding of ETS1 to the promoter region of Linc01056, while the level of Linc01056 increases with sorafenib treatment. **i.** Silencing ETS1 reduced the expression of LINC01056 under sorafenib induction. **j.** siETS1 reduced sorafenib sensitivity in the apoptosis assay. *p<0.05, **p<0.01

**
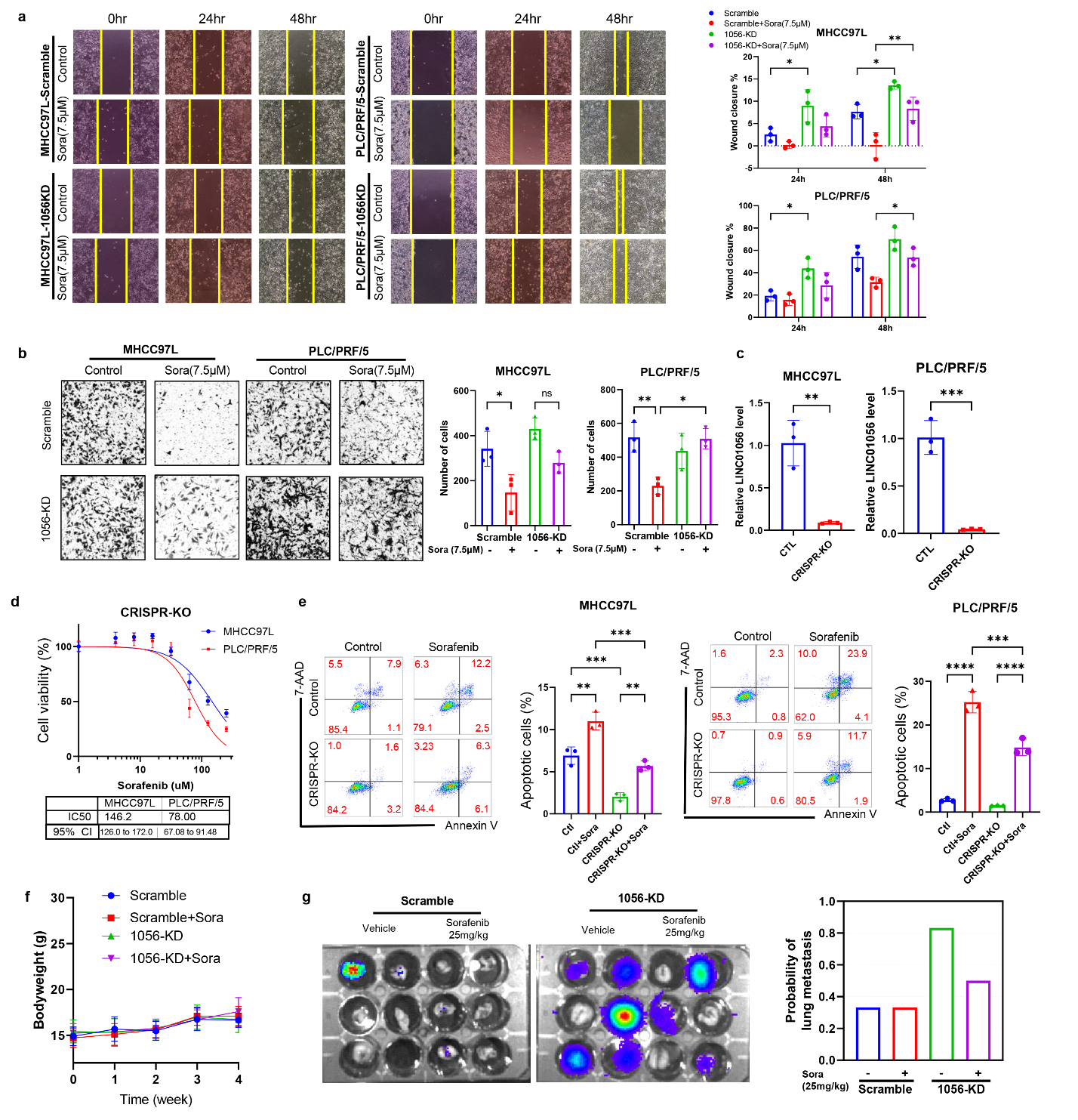
**

**Figure S2 Linc01056 knockdown induced in vitro invasion and in vivo metastasis of sorafenib-treated HCC. a.** Knockdown of Linc01056 increased the motility of HCC cells upon sorafenib treatment. **b.** Knockdown of Linc01056 increased the invasion of HCC cells through the Transwell in the presence of sorafenib. **c.** Linc01056 expression was knocked out by CRISPR genetic editing. **d.** CRISPR-KO of Linc01056 increased cell viability in sorafenib-treated HCC cells. **e.** CRISPR-KO of Linc01056 reduced sorafenib-induced apoptosis in HCC cells. **f.** Knockdown of Linc01056 had minimal effect on the body weight of sorafenib-treated mice. **g.** Knockdown of Linc01056 increase the lung metastasis of in vivo HCC cells in mice. *p<0.05, **p<0.01, ***p<0.001.

**
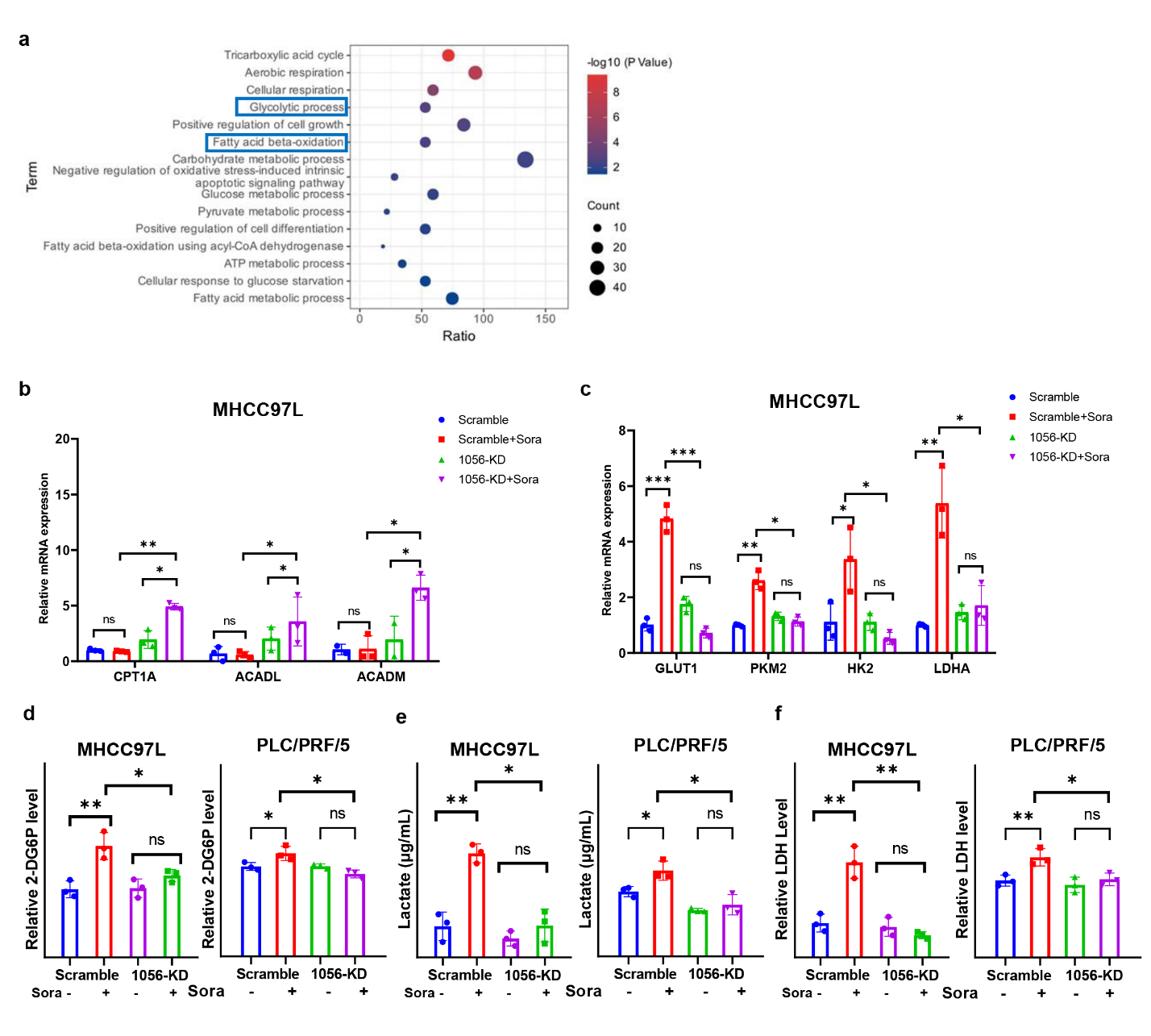
**

**Figure S3 Linc01056 knockdown induced FAO but suppressed glycolysis.** a. GO-BP enrichment on proteomics analysis of sorafenib-treated scramble or LINC01056-KD PLC/PRF/5 cells. **b.** Knockdown of Linc01056 significantly induced expression of FAO-related genes, but **c)** suppressed the induction of glycolysis-related genes by sorafenib in HCC cells. Knockdown of Linc01056 suppressed **d)** glucose uptake, **e)** release of lactic acid as well as **f)** the intracellular LDH activity in HCC cells**.** *p<0.05, **p<0.01, ***p<0.001

**
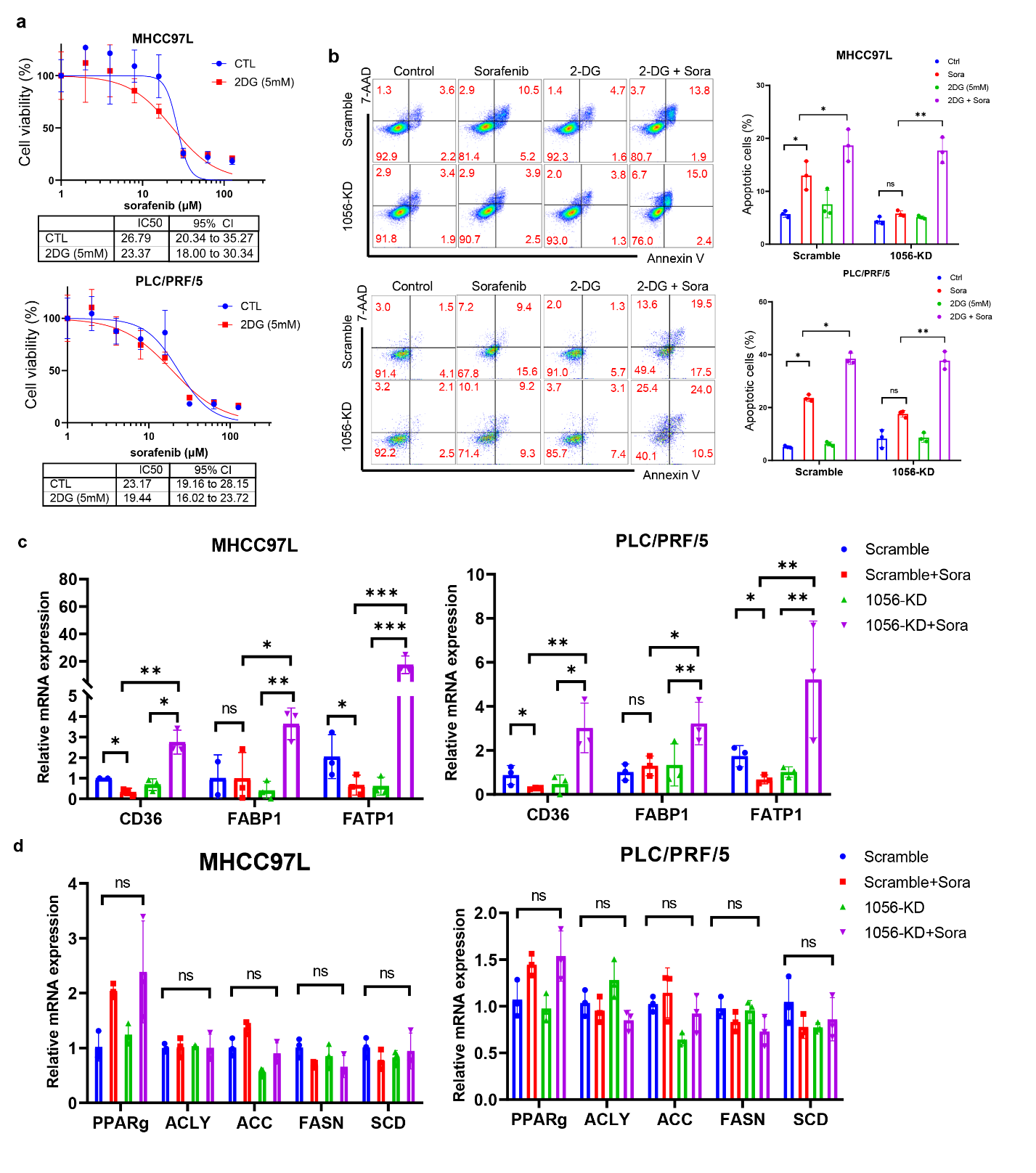
**

**Figure S4 inhibition of glycolysis and FAO improved sorafenib sensitivity in HCC cells with Linc01056 knockdown.** Suppression of sorafenib-induced glycolysis by 2-DG **a)** improved cytotoxicity of sorafenib and **b)** increased cell apoptosis induced by sorafenib in HCC cells. Linc01056 knockdown **c)** induced expression of fatty acid uptake-related genes, but not **d)** lipogenesis-related genes in HCC cells. *p<0.05, **p<0.01, ***p<0.001.


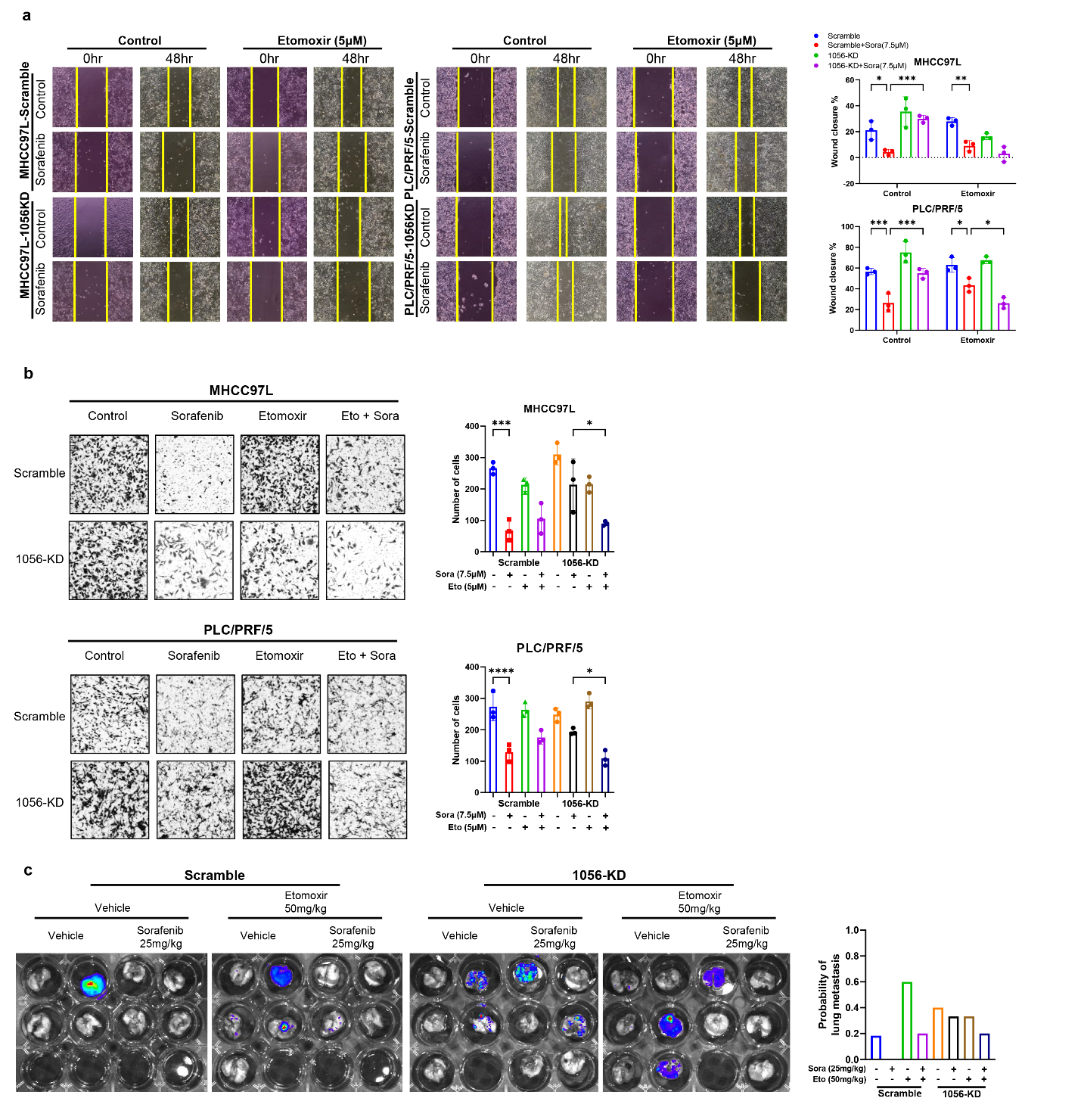


**Figure S5 Sorafenib sensitivity of HCC cells was improved by etomoxir.** FAO suppression by etomoxir **a)** reduced cell motility and **b)** invasion of in vitro HCC cells with Linc01056 knockdown, and **c)** suppressed lung metastasis of in vivo HCC. *p<0.05, **p<0.01, ***p<0.001


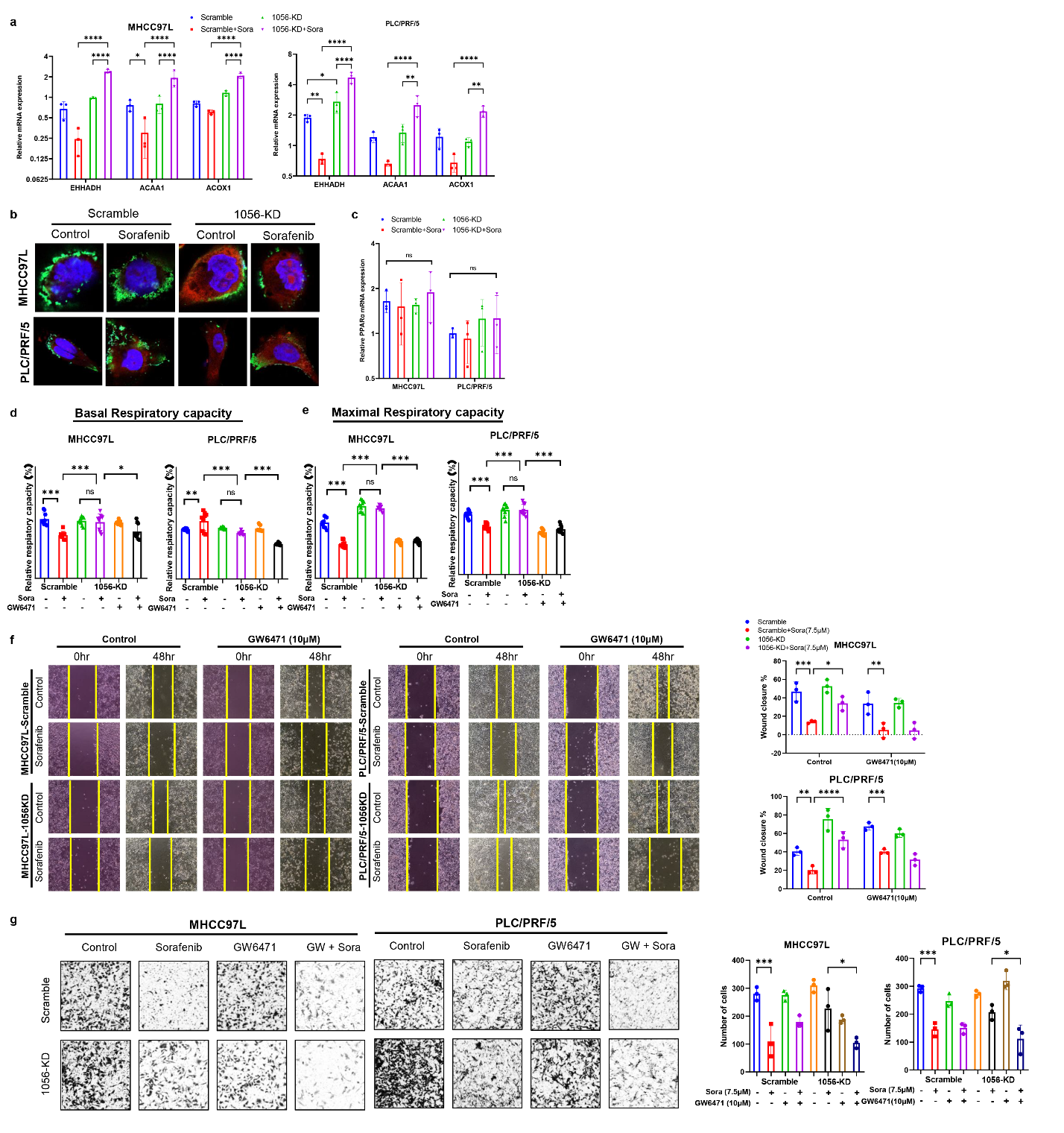


**Figure S6 Inhibition of FAO-sensitised Linc01056-knockdowned HCC cells to sorafenib treatment. a.** Linc01056 knockdown induced expression of FAO-related genes in HCC cells upon sorafenib exposure. **b.** Knockdown of Linc01056 induced the nuclear localization of PPARα. **c.** Linc01056 knockdown did not change the mRNA expression of PPARα. Suppression of PPARα activity by GW6471 reversed **d)** the increase of basal respiratory capacity and e**)** maximal respiratory capacity in Linc01056-knockdown HCC cells upon sorafenib treatment. GW6471 inhibited **f)** the increase of cell motility and **g)** invasion of Linc01056-knockdown HCC cells upon sorafenib treatment. *p<0.05, **p<0.01, ***p<0.001


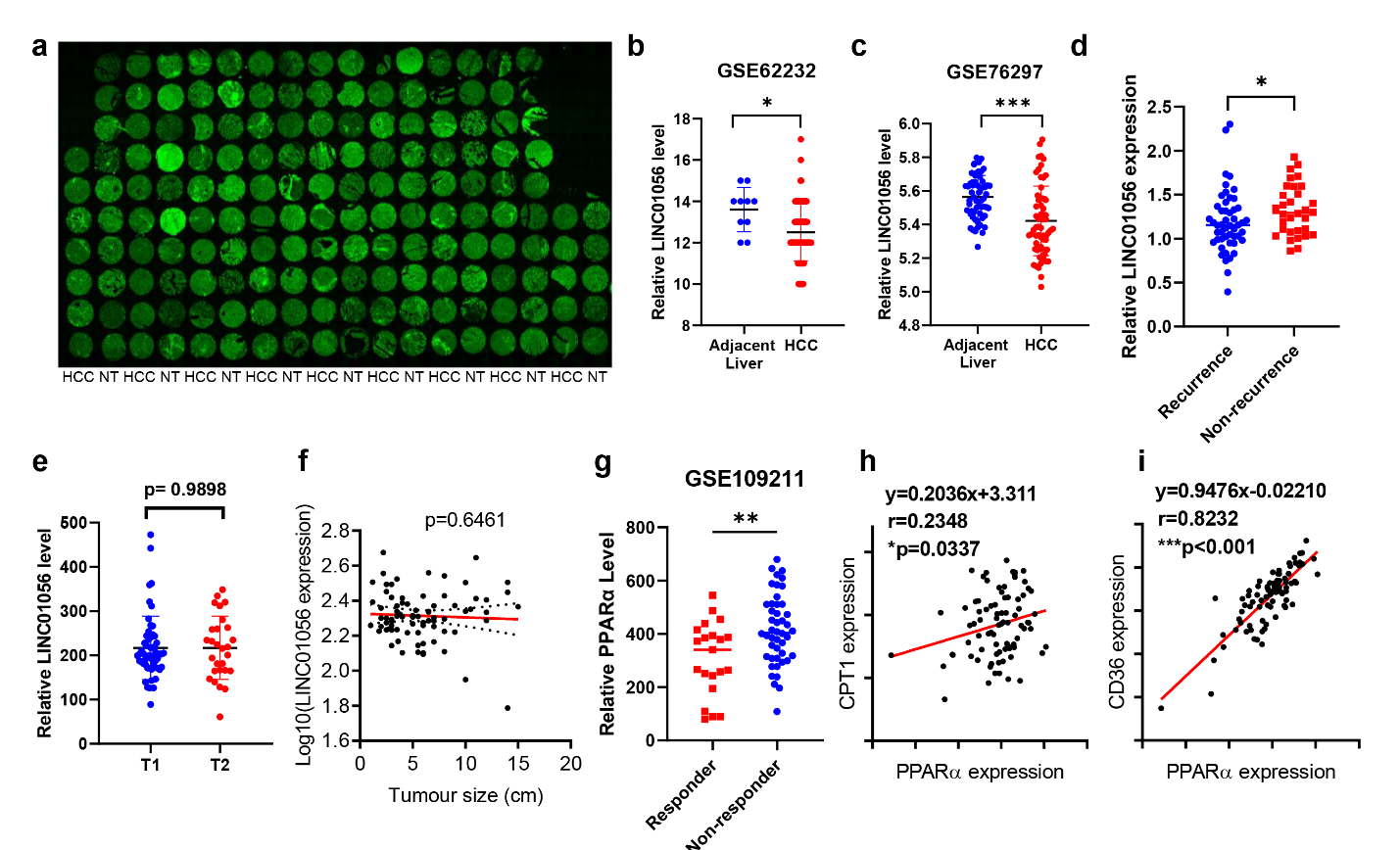


**Figure S7 The clinical significance of Linc01056 in HCC. a.** multiplex staining of tissue microarray of human HCC samples. Linc01056 expression was significantly lower in HCC compared to adjacent liver tissue in two data cohorts **b)** GSE62232 and **c)** GSE76297. **d.** Linc01056 expression was significantly lower in tumours of patients with recurrence. Linc01056 expression was not related to **e)** the TMN staging of HCC patients, or **f)** the size of tumours. **g.** PPARα expression level in the HCC tumour is higher in the sorafenib non-responder patients than the responder patients, data from GSE109211. Nuclear expression of PPARα was positively associated with **h)** CPT1, the marker of FAO, and **i)** CD36, the marker of fatty acid uptake. *p<0.05, ***p<0.001

**Supplemental Tables**

**Table S1 Sequence of Linc01056**

| >NR_033369.1 Homo sapiens long intergenic non-protein coding RNA 1056 (LINC01056), long non-coding RNA |
| --- |
| GAGGTCGCTCAGCACAGCGCTTCCAAAGTTATTTACCTGGTAGCGAGTGTCAGACTTCCTTCATTTTTCTGGCTGAATCCTATTCCCATGGGTGGATGGACCGCATTTTGTCCTCCATTCATCTGCTGGGGGCAACTGTGCTGTTTCCACCTTTCAGCCATTGTGAGTAACGCTGGTCTCTGGAGTGACAGGGATGGCCCTGCCCCACTGACCCAGGCGTTCACCCCAGAGAAGCGTGCAGGCAGGTACAAGAGGCCGAGTGCGGTCAACTTGAAGGGACAGGAAGCCTGCAGGCAGTGACCACTCTCTAGGCTGTGCTCAGAGGAGAGGCCAAGGGGACACAGGCACCAGGGCCGTGGGACACACCTGGGCTACCCCAGCTCCTATCAGGACTTGATGTGGCGTATGGAGCACTGACCGTCCATGTGGCAGGACTCAGAGGACGCGGGCAACACAGTGCCCAGGACAGAGAGGCCACAGAGGAGCAAACGGGCAGGCCGAGGACAGTCAAGACCAAGGGAAACGGCTAATTAATTAGGCTCAGCCTCTTGAAAGGCACTTAAAATACTAACGACTTGCTAAAAAAGGGCAGGACTTGAAAGCAAAGCCAAGGTCCAAAGTGCCCACCAGCGTGAGGGGTCTGCTGCCTCCCTGCGGGGAAGCCCTGGCAGAGCCCCTTGCTTTGCTGCTCACAGAGTCCAGGACATGAGAGCCGAGCCCATGCACCAGTGACTCACGCCCCGGCACAGATGAGCCCAGAGCCCGGAACAGAGGCCGTAGCAAGACCCCGGGAGCCCAGCCTCCCCTACCCCTCCAGCCCCGCTCTGGGCCCCAGGAGGCTGACCTCCATGGATGTCCCAGCAATCTGTCTCTGGCCTCTGGGCACTGGAAATACAGCGGTGGACACAGTGGTGGACCTCCCGGTATGTCCTCTGTTCGACTGAACAGTCGGGTTGCGGGGCCTCCAGGAGCTCTGTGAGCCCGTGCTCAGGTGCACACAACAGAAGTACTCCGCTGACGCCCACCCCTTTCACTCAGCTCTCGGTTCAGTCATCGCCAAGCAACTGAGAGCCTTTGTGAAGAGGCTTCCTGCCCTCTTCATCTTCCACAGACACGCTCTTCTTCCTTAAGCCCAGGCATACACAGACACAGTGAATGCACACACAAAGACACACACCAGTGTGCACACTGCTCTGGAATCCACAGGTTCTATAAATATCTAGAAATGATATCT |

**Table S2 Primers used in this study**

| **Gene** | **Forward primer (5’ to 3’)** | **Reverse primer (5’ to 3’)** |
| --- | --- | --- |
| LINC01056 | CAAGACCAAGGGAAACGGCT | CTGGACTCTGTGAGCAGCAAA |
| MALAT1 | GACGGAGGTTGAGATGAAGC | ATTCGGGGCTCTGTAGTCCT |
| LINC01523 | ACCCAGGAGACAGGAGGATT | GCAGCTCAGAGAGCCACTTT |
| GATA3-AS1 | CGGGGTATGTGTGTCCTCTT | TCTGTACCCGACTGGGTTTC |
| Lnc-ANKRD20A4-12 | TGAGTGGCTCTGTGTGAAGG | GCAGGATATGGCAAGGATGT |
| Lnc-CELA3A-5 | CACAGAGATGTCAGCCTGGA | ATAAAGCTGCCTCAGCCAAA |
| CPT1 | TCCAGTTGGCTTATCGTGGTG | TCCAGAGTCCGATTGATTTTTGC |
| ACADL | TGCAATAGCAATGACAGAGCC | CGCAACTACAATCACAACATCAC |
| ACADM | TGGATAACCAACGGAGGAAAAG | CTGGGGTATCTGCTTCCACA |
| GLUT1 | TCTGGCATCAACGCTGTCTTC | CGATACCGGAGCCAATGGT |
| PKM2 | ATGTCGAAGCCCCATAGTGAA | TGGGTGGTGAATCAATGTCCA |
| HK2 | TGCCACCAGACTAAACTAGACG | CCCGTGCCCACAATGAGAC |
| LDHA | TTGACCTACGTGGCTTGGAAG | GGTAACGGAATCGGGCTGAAT |
| CD36 | GGCTGTGACCGGAACTGTG | AGGTCTCCAACTGGCATTAGAA |
| FABP1 | GTGTCGGAAATCGTGCAGAAT | GACTTTCTCCCCTGTCATTGTC |
| FATP1 | GGGGCAGTGTCTCATCTATGG | CCGATGTACTGAACCACCGT |
| PPARg | TACTGTCGGTTTCAGAAATGCC | GTCAGCGGACTCTGGATTCAG |
| ACLY | TCGGCCAAGGCAATTTCAGAG | CGAGCATACTTGAACCGATTCT |
| ACC | ATGTCTGGCTTGCACCTAGTA | CCCCAAAGCGAGTAACAAATTCT |
| FASN | AAGGACCTGTCTAGGTTTGATGC | TGGCTTCATAGGTGACTTCCA |
| SCD | TCTAGCTCCTATACCACCACCA | TCGTCTCCAACTTATCTCCTCC |
| EHHADH | TCCTGTGATTGCTGTAGACTCG | GGCCGCTCTGTTGCATTTTG |
| ACAA1 | TGTGGAGAAGCTACGACTCCC | CACCACTCCGTATGCCCTC |
| ACOX1 | ACTCGCAGCCAGCGTTATG | AGGGTCAGCGATGCCAAAC |
| GAPDH | ACAACTTTGGTATCGTGGAAGG | GCCATCACGCCACAGTTTC |
| β-actin | CATGTACGTTGCTATCCAGGC | CTCCTTAATGTCACGCACGAT |

**Table S2 Patient information**

| **Sample code** | **Sex** | **Age** | **Size of tumour** | **Number of tumour** | **Survival status** | **Overall survival (month)** | **Progression-free survival (month)** | **T** | **N** | **M** | **Recurrence** | **Cirrhosis** | **HBsAg** | **HBcAb** | **AntiHCV** | **TB（umol/L）** | **ALT（U/L）** | **ALB（g/dl）** | **AFP（ug/L）** | **GGT（U/L）** |
| --- | --- | --- | --- | --- | --- | --- | --- | --- | --- | --- | --- | --- | --- | --- | --- | --- | --- | --- | --- | --- |
| D19A3427 | F | 73 | 4 | 1 | 1 | 51 | 48 | T1 | N0 | M0 | Y | + | + | + | - | 11.5 | 41 | 4 | 6 | 37 |
| D19A3428 | F | 37 | 1.6 | 1 | 0 | 72 | 72 | T1 | N0 | M0 | N | + | + | + | - | 6.6 | 16 | 4.4 | 49 | 11 |
| D19A3429 | M | 53 | 4.5 | 3 | 1 | 34 | 12 | T1 | N0 | M0 | Y | + | + | + | - | 10.8 | 65 | 3.9 | 6 | 215 |
| D19A3430 | M | 41 | 7 | 2 | 0 | 71 | 5 | T2 | N0 | M0 | Y | + | + | + | - | 9.9 | 59 | 4.6 | 2128 | 79 |
| D19A3431 | F | 80 | 7 | 1 | 1 | 23 | 20 | T2 | N0 | M0 | Y | - | - | - | - | 13.7 | 55 | 3.7 | 2 | 157 |
| D19A3432 | M | 56 | 1.8 | 1 | 0 | 71 | 71 | T1 | N0 | M0 | N | + | + | + | - | 22.2 | 62 | 3.7 | 169 | 78 |
| D19A3433 | M | 41 | 3 | 1 | 1 | 53 | 15 | T1 | N0 | M0 | Y | + | + | + | - | 18.3 | 48 | 5 | 34 | 47 |
| D19A3434 | M | 52 | 4 | 1 | 0 | 70 | 9 | T2 | N0 | M0 | Y | + | + |  |  | 23 | 182 | 4 | 35 | 61 |
| D19A3435 | M | 49 | 4.5 | 1 | 1 | 47 | 29 | T2 | N0 | M0 | Y | + | + | + | - | 7.8 | 50 | 4.2 | 98 | 50 |
| D19A3436 | M | 50 | 5.5 | 2 | 1 | 19 | 19 | T2 | N0 | M0 | N | + | + | + | - | 27 | 62 | 5 | 689 | 138 |
| D19A3437 | F | 43 | 3 | 1 | 0 | 69 | 69 | T1 | N0 | M0 | N | + | + | + | - | 9.6 | 556 | 5 | 246 | 18 |
| D19A3438 | M | 63 | 4 | 1 | 1 | 57 | 51 | T1 | N0 | M0 | Y | + | + | + | - | 11.4 | 42 | 4.6 | 783 | 27 |
| D19A3439 | M | 43 | 7 | 1 | 0 |  | 50 | T1 | N0 | M0 | Y | + | + | + | - | 24.8 | 393 | 3.9 | 13060 | 73 |
| D19A3440 | M | 38 | 7 | 1 | 1 | 19 | 12 | T1 | N0 | M0 | Y | + | + | + | - | 13.6 | 44 | 4.3 | 63 | 211 |
| D19A3441 | M | 45 | 12 | 4 | 1 | 13 | 4 | T1 | N0 | M0 | Y | + | + | + | - | 17 | 84 | 3.6 | 225 | 184 |
| D19A3442 | F | 59 | 8 | 1 | 1 | 40 | 16 | T2 | N0 | M0 | Y | - | + | + | - | 16.6 | 40 | 3.6 | 21315 | 141 |
| D19A3443 | M | 56 | 8 | 1 | 0 | 68 | 68 | T1 | N0 | M0 | N | + | + | + | - | 10.7 | 52 | 4.7 | 4202 | 70 |
| D19A3444 | M | 46 | 3.5 | 1 | 0 | 67 | 67 | T2 | N0 | M0 | N | + | + | + | - | 8.8 | 26 | 5.2 | 2 | 24 |
| D19A3445 | M | 27 | 11 | 1 | 1 | 41 | 32 | T2 | N0 | M0 | Y | + | + | + | - | 10.2 | 61 | 4.9 | 60500 | 63 |
| D19A3446 | M | 54 | 3.5 | 1 | 1 | 20 | 7 | T1 | N0 | M0 | Y | - | + | + | - | 10 | 65 | 4.2 | 642 | 110 |
| D19A3447 | M | 50 | 2.8 | 2 | 0 | 65 | 25 | T1 | N0 | M0 | Y | + | + | + | - | 8.4 | 43 | 4.8 | 6 | 18 |
| D19A3448 | M | 59 | 12 | 1 | 0 | 65 | 65 | T2 | N0 | M0 | N | + | - | - | - | 12.1 | 22 | 4.6 | 3 | 71 |
| D19A3449 | M | 59 | 12 | 1 | 1 | 17 | 6 | T2 | N0 | M0 | Y | + | + | + | - | 16.2 | 75 | 3.8 | 60500 | 277 |
| D19A3450 | M | 44 | 4 | 1 | 0 | 65 | 65 | T1 | N0 | M0 | N | + | + | + | - | 22 | 76 | 5 | 2 | 170 |
| D19A3451 | M | 39 | 5 | 1 | 1 | 10 | 5 | T1 | N0 | M0 | Y | + | + |  |  | 19.5 | 38 | 5.3 | 3000 | 99 |
| D19A3452 | F | 70 | 10.5 | 1 | 0 | 65 | 65 | T2 | N0 | M0 | N | + | - | - | - | 24.3 | 49 | 4 | 232 | 74 |
| D19A3453 | M | 51 | 3 | 1 | 1 | 31 | 31 | T1 | N0 | M0 | N | - | + | + | - | 11.4 | 77 | 4.1 | 9 | 47 |
| D19A3454 | M | 54 | 9 | 1 | 1 | 13 | 5 | T1 | N0 | M0 | Y | + | + | + | - | 39.1 | 395 | 3.4 | 21200 | 105 |
| D19A3455 | M | 48 | 9 | 1 | 1 | 7 | 5 | T2 | N0 | M0 | Y | + | + | + | - | 17 | 34 | 4.4 | 495 | 77 |
| D19A3456 | M | 35 | 2.5 | 3 | 1 | 48 | 12 | T2 | N0 | M0 | Y | + | + | + | - | 15.4 | 41 | 3.8 | 18 | 285 |
| D19A3457 | M | 53 | 7 | 1 | 1 | 13 | 10 | T1 | N0 | M0 | Y | + | + | + | - | 22.6 | 48 | 3.9 | 7070 | 68 |
| D19A3458 | M | 48 | 5 | 3 | 1 | 16 | 16 | T1 | N0 | M0 | Y | + | + | + | - | 20 | 36 | 3.6 | 11 | 53 |
| D19A3459 | M | 46 | 2.5 | 1 | 0 | 63 | 53 | T1 | N0 | M0 | Y | + | + | + | - | 15.4 | 92 | 4.9 | 5 | 75 |
| D19A3460 | M | 29 | 3 | 1 | 0 |  |  | T1 | N0 | M0 | N | + | + | + | - | 9.3 | 36 | 3.9 | 40 | 30 |
| D19A3461 | M | 62 | 5.5 | 2 | 0 | 62 | 62 | T1 | N0 | M0 | N | + | + | + | - | 15.2 | 30 | 4.6 | 72 | 56 |
| D19A3462 | M | 57 | 4 | 1 | 0 | 61 | 61 | T1 | N0 | M0 | N | - | + | + | - | 15.7 | 31 | 4.3 | 426 | 48 |
| D19A3463 | M | 79 | 14 | 1 | 1 | 15 | 9 | T2 | N0 | M0 | Y | + | - | - | - | 6.8 | 15 | 4.9 | 3 | 165 |
| D19A3464 | M | 39 | 5.5 | 2 | 0 | 60 | 60 | T1 | N0 | M0 | N | + | + | + | - | 14.8 | 50 | 5 | 161 | 131 |
| D19A3465 | M | 39 | 6.5 | 2 | 1 | 22 | 5 | T1 | N0 | M0 | Y | + | + | + | - | 11.3 | 31 | 4.1 | 119 | 63 |
| D19A3466 | F | 55 | 8 | 1 | 0 | 60 | 60 | T1 | N0 | M0 | N | + | - | - | - | 7.6 | 26 | 5.7 | 1 | 136 |
| D19A3467 | M | 59 | 2.5 | 1 | 0 | 60 | 45 | T1 | N0 | M0 | Y | + | + | + | - | 24.6 | 17 | 3.9 | 573 | 51 |
| D19A3468 | F | 60 | 1 | 2 | 0 | 60 | 56 | T1 | N0 | M0 | Y | + | + | + | - | 8.9 | 24 | 4.4 | 435 | 19 |
| D19A3469 | M | 59 | 2 | 1 | 0 | 60 | 60 | T1 | N0 | M0 | N | - | + | + | - | 23.9 | 32 | 3.7 | 334 | 38 |
| D19A3470 | M | 75 | 3.5 | 1 | 1 | 9 | 7 | T1 | N0 | M0 | Y | - | + | + | - | 18.6 | 43 | 4 | 10 | 190 |
| D19A3471 | M | 66 | 3.5 | 1 | 1 | 37 | 37 | T2 | N0 | M0 | N | - | + | + | - | 18.9 | 29 | 3.8 | 4 | 26 |
| D19A3472 | M | 34 | 1.2 | 1 | 0 | 60 | 60 | T1 | N0 | M0 | N | + | + | + | - | 16.7 | 41 | 4.6 | 240 | 48 |
| D19A3473 | M | 42 | 6 | 1 | 1 | 27 | 15 | T2 | N0 | M0 | Y | + | + | + | - | 13.1 | 49 | 4.5 | 13 | 152 |
| D19A3474 | M | 41 | 4.5 | 1 | 1 | 13 | 7 | T1 | N0 | M0 | Y | + | + | + | - | 12.7 | 62 | 4.9 | 1110 | 104 |
| D19A3475 | M | 57 | 8 | 1 | 1 | 12 | 12 | T2 | N0 | M0 | N | + | - | + | - | 9.2 | 81 | 4.7 | 214 | 55 |
| D19A3476 | M | 59 | 10 | 1 | 1 | 24 | 13 | T2 | N0 | M0 | Y | + | - | - | - | 7.3 | 95 | 3.8 | 4 | 56 |
| D19A3477 | M | 51 | 3.5 | 1 | 0 | 59 | 12 | T1 | N0 | M0 | Y | - | + | + | - | 19.7 | 62 | 4.2 | 1511 | 27 |
| D19A3478 | M | 84 | 5.5 | 1 | 1 | 16 | 12 | T2 | N0 | M0 | Y | + | + | - | - | 10.3 | 47 | 3.9 | 19 | 25 |
| D19A3479 | M | 39 | 6.5 | 1 | 0 | 59 | 4 | T1 | N0 | M0 | Y | + | + | + | - | 7.8 | 51 | 5 | 3302 | 45 |
| D19A3480 | M | 60 | 2.2 | 1 | 1 | 47 | 42 | T1 | N0 | M0 | Y | + | + | + | - | 5.8 | 22 | 4.9 | 131 | 40 |
| D19A3481 | M | 40 | 11 | 1 | 0 | 59 | 7 | T1 | N0 | M0 | Y | + | + | + | - | 10.5 | 45 | 5.3 | 4031 | 86 |
| D19A3482 | M | 35 | 2.5 | 1 | 1 | 12 | 12 | T1 | N0 | M0 | N | - | + | + | - | 15.7 | 74 | 4.3 | 67 | 18 |
| D19A3483 | F | 60 | 6.5 | 1 | 0 | 59 | 59 | T1 | N0 | M0 | N | + | + | + | - | 13 | 29 | 4.5 | 1 | 65 |
| D19A3484 | M | 56 | 6.5 | 2 | 1 | 44 | 37 | T1 | N0 | M0 | Y | + | + | + | - | 18.2 | 96 | 5.3 | 44295 | 208 |
| D19A3485 | M | 50 | 3 | 1 | 0 | 59 | 32 | T1 | N0 | M0 | Y | + | + | + | - | 11 | 66 | 4.9 | 22 | 51 |
| D19A3486 | M | 31 | 8 | 2 | 1 | 38 | 38 | T1 | N0 | M0 | N | + | + | + | - | 15 | 29 | 4.2 | 60500 | 75 |
| D19A3487 | M | 63 | 14 | 1 | 1 | 29 | 25 | T2 | N0 | M0 | Y | + | + | + | - | 15.8 | 21 | 4.3 | 557 | 99 |
| D19A3488 | F | 42 | 2 | 1 | 0 | 59 | 59 | T1 | N0 | M0 | N | + | + | + | - | 11.8 | 21 | 3.8 | 304 | 24 |
| D19A3489 | M | 47 | 4 | 1 | 1 | 12 | 12 | T1 | N0 | M0 | N | + | - | + | - | 12.6 | 835 | 3.8 | 39205 | 19 |
| D19A3490 | M | 58 | 3 | 1 | 1 | 63 | 13 | T2 | N0 | M0 | Y | + | + | + | - | 16.8 | 132 | 3.8 | 50 | 18 |
| D19A3491 | F | 70 | 4.5 | 1 | 0 | 59 | 59 | T2 | N0 | M0 | N | + | - | - | - | 6.7 | 8 | 4.5 | 151 | 10 |
| D19A3492 | M | 41 | 3.8 | 1 | 0 | 58 | 58 | T1 | N0 | M0 | N | - | + | + | - | 17.3 | 30 | 4.3 | 13 | 60 |
| D19A3493 | M | 72 | 7 | 1 | 0 | 58 | 58 | T1 | N0 | M0 | N | + | + | + | - | 16.3 | 13 | 4.1 | 0 | 45 |
| D19A3494 | M | 64 | 6 | 1 | 1 | 13 | 8 | T1 | N0 | M0 | Y | + | - | + | - | 5.9 | 35 | 4 | 1 | 168 |
| D19A3495 | M | 49 | 2 | 1 | 0 | 58 | 58 | T1 | N0 | M0 | N | - | + | + | - | 21.4 | 179 | 4.5 | 4 | 112 |
| D19A3496 | M | 50 | 10 | 1 | 1 | 48 | 7 | T1 | N0 | M0 | Y | + | - | + | - | 22.9 | 23 | 4.6 | 25600 | 186 |
| D19A3497 | F | 72 | 6 | 1 | 1 | 39 | 6 | T2 | N0 | M0 | Y | + | + | + | - | 21.6 | 29 | 4.1 | 7955 | 25 |
| D19A3498 | M | 54 | 1.2 | 1 | 0 | 58 | 58 | T1 | N0 | M0 | N | + | + | + | - | 14.1 | 60 | 4.3 | 36 | 63 |
| D19A3499 | M | 39 | 10 | 1 | 1 | 20 | 13 | T2 | N0 | M0 | Y | + | + | + | - | 7 | 28 | 4.8 | 60500 | 169 |
| D19A3500 | M | 48 | 3 | 1 | 1 | 41 | 38 | T2 | N0 | M0 | Y | + | + | + | - | 13.9 | 96 | 3.4 | 337 | 86 |
| D19A3501 | M | 60 | 5.3 | 1 | 0 | 58 | 58 | T1 | N0 | M0 | N | + | + | + | - | 15.5 | 21 | 4.7 | 1 | 27 |
| D19A3502 | F | 56 | 5 | 1 | 1 | 51 | 19 | T1 | N0 | M0 | Y | + | + | + | - | 16.5 | 34 | 5.5 | 6580 | 49 |
| D19A3503 | F | 45 | 2.2 | 1 | 0 | 57 | 57 | T1 | N0 | M0 | N | + | + | + | - | 6.5 | 16 | 4.8 | 208 | 23 |
| D19A3504 | M | 55 | 6 | 1 | 0 | 57 | 57 | T1 | N0 | M0 | N | + | + | + | - | 13.3 | 31 | 4.7 | 5 | 42 |
| D19A3505 | M | 43 | 2.5 | 2 | 1 | 17 | 10 | T1 | N0 | M0 | Y | + | + | + | - | 9.1 | 48 | 4 | 2169 | 81 |
| D19A3506 | F | 64 | 6 | 1 | 1 | 27 | 21 | T1 | N0 | M0 | Y | + | + | + | - | 21.9 | 33 | 4.6 | 19920 | 37 |
| D19A3507 | M | 51 | 2 | 1 | 1 | 39 | 16 | T1 | N0 | M0 | Y | + | - | - | + | 16 | 120 | 3.6 | 50 | 110 |
| D19A3508 | M | 69 | 5 | 1 | 1 | 41 | 30 | T1 | N0 | M0 | Y | + | + | + | - | 13.2 | 37 | 4.4 | 77 | 37 |
| D19A3509 | F | 62 | 5.5 | 2 | 1 | 41 | 21 | T1 | N0 | M0 | Y | + | + | + | - | 19.6 | 23 | 4.6 | 8 | 49 |
| D19A3510 | M | 45 | 4 | 1 | 0 | 57 | 57 | T1 | N0 | M0 | N | + | + | + | - | 8.4 | 39 | 5.1 | 1880 | 110 |
| D19A3511 | M | 58 | 3 | 1 | 0 | 57 | 57 | T2 | N0 | M0 | N | + | + | + | - | 32 | 28 | 5 | 3144 | 42 |
| D19A3512 | M | 64 | 3.6 | 1 | 0 | 57 | 57 | T2 | N0 | M0 | N | + | - | + | - | 12.8 | 31 | 5 | 112 | 110 |
| D19A3513 | M | 42 | 6 | 1 | 0 | 57 | 57 | T1 | N0 | M0 | N | + | + | + | - | 15 | 19 | 4.7 | 3 | 23 |
| D19A3514 | M | 49 | 15 | 1 | 1 | 8 | 6 | T2 | N0 | M0 | Y | + | + | + | - | 10.5 | 62 | 4.9 | 132 | 648 |
| D19A3515 | M | 41 | 2 | 1 | 0 | 57 | 57 | T1 | N0 | M0 | N | + | + | + | - | 7.4 | 14 | 5.3 | 54 | 29 |
| D19A3516 | M | 55 | 14 | 2 | 1 | 10 | 7 | T2 | N0 | M0 | Y | + | + | + | - | 10.3 | 20 | 4.4 | 2051 | 156 |

**Supplemental Materials and Methods**

## Linc01056-KD Cell Line

LncRNA knockdown was performed with commercially obtained short hairpin RNA (shRNA) pairs from IGE Biotechnology (China). The target of sh-1056-1 is ACCAAGGGAAACGGCTAATTA and sh-1056-2 is AGTGTCAGACTTCCTTCATTT. Plasmids were packaged using the lentiviral vectors pRSV-Rev and pMDLg/pRRE, which were gifts from Didier Trono (Addgene plasmid #12251 and #12253; <http://n2t.net/addgene:12253;> RRID: Addgene_12,253)[[2](#_ENREF_2)], and the envelope plasmid pCMV-VSV-G, which was a gift from Bob Weinberg (Addgene plasmid #8454; <http://n2t.net/addgene:8454;> RRID: Addgene_8454)[[3](#_ENREF_3)], in HEK293FT cells using Lipofectamine 3000 (Invitrogen, USA) for transfection. The culture medium was collected after 48 h and 72 h and then applied to transduce MHCC97L or PLC/PRF/5 cells after passage through a 0.45 μm filter. Stably transduced cells were selected with 0.3 μg/mL puromycin for seven days, and knockdown was validated by quantitative real-time PCR (qPCR). A rescue assay was performed by transfecting a pCDH-CMV-MCS-EF1α plasmid carrying the whole segment of Linc01056 into the Linc01056-KD cell line. The expression level of Linc01056 was measured by qPCR.

## Proteomics Analysis

Cells were cultured in 10 cm dishes and treated with sorafenib for 24 h. The cells were harvested at 80-90% confluence and were then subjected to proteomics analysis at the Proteomics and Metabolomics Core Facility, LKS Faculty of Medicine, the University of Hong Kong. Proteins were extracted in EasyPep lysis buffer (100 μL per min, 1 million cells) with universal nuclease. The protein concentration in the lysate was then quantified using a BCA assay (Thermo Pierce), and 100 μg of protein from each sample was then subjected to reduction and alkylation at 95°C for 10 min. After cooling to room temperature, the proteins were subjected to LysC/trypsin digestion at 37°C for 120 min. Digestion was stopped by acidification, and the peptides were washed and desalted using the spin column provided in the kit before they were analysed by liquid chromatography–tandem mass spectrometry (LC‒MS/MS). Eluted peptides were analysed with a nanoElute UHPLC system coupled to a Bruker timsTOF Pro mass spectrometer. The peptide mixture was loaded onto an Aurora C18 UHPLC column (75 μm i.d. × 25 cm length × 1.6 μm particle size (IonOpticks, Australia)). Chromatographic separation was carried out using a linear gradient of 2-30% buffer B (0.1% formic acid (FA) in acetonitrile (ACN)) at a flow rate of 250 nL/min over 100 min. MS data were collected over a m/z range of 100 to 1700 and an MS/MS range of 100 to 1700. During MS/MS data collection, each TIMS cycle was 1.1 s and included 1 MS plus an average of 10 parallel accumulation–serial fragmentation (PASEF) MS/MS scans. Raw mass spectrometry data were processed using MaxQuant version 1.6.14.0, wherein the Andromeda algorithm was used to search the data against the Human SwissProt FASTA database (May 2022) containing 20,361 entries. The following search settings were used: oxidized methionine (M) and acetylation (Protein N-term) were selected as dynamic modifications, and carbamidomethyl (C) was selected as the fixed modification with a minimum peptide length of 7 amino acids. High-confidence proteins were identified using a target-decoy approach with a reverse database and a strict false discovery rate of 1% at the peptide and PSM levels. Proteins identified under both analysis conditions were quantified using the peptide label-free quantitation (LFQ) intensities, and their ratios were used for label-free quantitation to calculate the fold change values. Data visualization and statistical data analysis were performed by Perseus software version 1.6.13.0.

## Fatty Acid Metabolomics

Sample processing and gas chromatography‒mass spectrometry (GC‒MS) analysis were performed at the Centre for PanorOmic Sciences - Proteomics and Metabolomics Core, LKS Faculty of Medicine, University of Hong Kong. For sample homogenization and metabolite extraction, 100 µL of chloroform with 20 µg of C19:0 fatty acid internal standard was spiked into the sample. The sample was extracted with 5 rounds of 2:1 chloroform/methanol incubation followed by sonication. After centrifugation, the supernatant was further cleaned by liquid‒liquid extraction in 0.73% NaCl and methanol. The resultant mixture was dried under an N2 stream at 45°C before transesterification. One millilitre of methanol and 50 µL of concentrated hydrochloric acid (35%, w/w) were then added to the sample. The solution was overlaid with nitrogen, and the tube was tightly closed. After vortexing, the tube was heated at 100°C for 1.5 h. After cooling to room temperature, 1 mL of hexane and 1 mL of water were added for fatty acid methyl ester (FAME) extraction. The tube was vortexed, and after phase separation, 1 µL of the hexane phase was injected for GC‒MS analysis. GC–MS chromatograms were acquired in scan and selected ion monitoring (SIM) mode in an Agilent 7890B GC - Agilent 7010 Triple Quadrupole Mass Spectrometer system. The samples were separated on Agilent DB-23 capillary columns (60 m × 0.25 mm ID, 0.15 µm film thickness) under helium at a constant pressure of 33.4 psi. The GC oven program started at 50°C (hold time 1 min), and the oven temperature was increased to 175°C at a ramp rate of 25°C/min. The temperature was then raised to 190°C (hold time 5 min) at a ramp rate of 3.5°C/min. Finally, the temperature was raised to 220°C (hold time 4 min) at a ramp rate of 2°C/min. The inlet temperature and transfer line temperature were 250°C and 280°C, respectively. Characteristic fragment ions (m/z 55, 67, 69, 74, 79, 81, 83, 87, 91, 93, 95, 96, 97, 115, 127, 143) were monitored in SIM mode throughout the run. Mass spectra from m/z 50-350 were acquired in scan mode.

## Chromatin Immunoprecipitation (ChIP)-qPCR

ChIP was performed using the EZ-Magna ChIP A/G Chromatin Immunoprecipitation kit (Merck Millipore, Germany) following the manufacturer’s instructions. Cells were fixed with 1% PFA and were then harvested for nuclear separation. Nuclear lysates were sheared by sonication under optimal conditions (7 s pulse on, 10 s pulse off, 15 cycles, 40% amplitude) to yield DNA fragments of 200–700 base pairs (bp). Ten percent of the lysate was aliquoted as input, and the remaining lysate was subjected to immunoprecipitation with specific antibody- or IgG control-coated magnetic beads. DNA was then purified and analysed by qPCR.

## RNA Immunoprecipitation (RIP)

RIP was performed with a protocol modified from the literature [[4](#_ENREF_4)]. In brief, MHCC97L cells were grown and collected at 80-90% confluence. A total of 5 × 10^6^ cells were counted and fixed using 1% formaldehyde solution. After washing with PBS, the cells were resuspended in NP-40 lysis buffer and frozen at -80°C overnight. An anti-PPARα antibody (Invitrogen) or control IgG (Invitrogen) was mixed with protein A/G magnetic beads at a ratio of 1:20, and the mixture was incubated with rotation. On the next day, the magnetic beads were washed five times with 0.2% Tween 20 in PBS. Ten percent of the cell lysate was aliquoted and saved as input. The remaining sample was mixed with the magnetic beads and incubated with rotation at 4°C overnight. After washing, the magnetic beads were resuspended in proteinase K buffer and incubated at 55°C for 30 min with shaking. The phenol‒chloroform-isoamyl alcohol method was used to purify RNA. The RNA content was measured by qPCR.

## Oxygen Consumption Rate (OCR)/Extracellular Acidification Rate (ECAR) Assay

The OCR and ECAR were measured on the Seahorse XFe96 platform (Agilent, USA) according to the protocol. In brief, 2.5×10^4^ cells were seeded into each well of a 96-well plate and treated with the desired drug. On the day of the assay, the cells were washed with XF assay medium once and then incubated in a non-CO2 incubator for one hour. For ECAR measurement, 2 mM glutamine was added to phenol red-free DMEM, while for OCR measurement, extra 1 mM sodium pyruvate and 10 mM glucose were added. Measurement of OCR was performed by sequential injection of 1 μM oligomycin (Sigma‒Aldrich), 1.5 μM FCCP (Sigma‒Aldrich), and 1 μM rotenone (Sigma‒Aldrich) + 1 μM antimycin A (Sigma‒Aldrich). Measurement of ECAR was performed by sequential injection of 10 mM glucose (Sigma‒Aldrich), 1 μM oligomycin (Sigma‒Aldrich), and 50 mM 2-deoxy-D-glucose (2-DG; Sigma‒Aldrich). The time interval between each measurement was 6 min. Basal respiration and maximal respiration rates were calculated with average data points from the graph.

## qPCR

Total RNA was extracted using RNAiso Plus reagent (Takara, Japan). Reverse transcription was performed using a HiScript III 1st Strand cDNA Synthesis Kit (Vazyme, China) following the manufacturer’s instructions. Real-time PCR was performed with ChamQ SYBR Colour qPCR Master Mix (Vazyme) on a LightCycler 480 (Roche, Switzerland). The list of primer sets is included in Supplementary Table S2.

## Cell Viability Assay

Cells (5,000/well) were seeded into 96-well plates the day before the assay was performed. Sorafenib or other inhibitors were added to the culture medium and applied for the desired time. Cell viability was determined by the MTT method by adding 0.5 mg/mL MTT (Sigma‒Aldrich, USA) for a 4 h incubation. The absorbance was measured at 490 nm using a Multiskan MS microplate reader (Labsystems, Finland).

## Colony Formation Assay

Two thousand MHCC97L or PLC/PRF/5 cells were seeded in a 6-well plate and incubated until colonies formed. Cells were cultured in complete DMEM. The cells were washed with PBS and then fixed with 4% PFA. The colonies were then stained with 0.1% crystal violet (Sigma‒Aldrich, USA) for visualization and imaged with illumination.

## Cell Migration Assay

The cell migration ability was measured by a wound healing assay. In brief, cells were seeded in 12-well plates and grown until 90-100% confluent. Gaps with a consistent width were made in the cell layer by scraping using sterile 1000 μL pipette tips. The culture dishes were washed with PBS three times, and the remaining cells were then cultured with serum-free DMEM. The wounds were photographed immediately, 24 h and 48 h after scratching.

## Cell Invasion Assay

Matrigel basement membrane was used to coat Transwell system membranes with a pore size of 8.0 μm (Corning, USA) by incubation for 45 min at 37°C before cell seeding. A total of 1×10^4^ cells resuspended in 100 μL of basal medium without serum were added to the upper chambers, while 600 μL complete DMEM supplemented with 10% FBS was added to the lower chambers. The Transwell inserts were washed with PBS and fixed with 4% PFA after 24 h. The inserts were then stained with 0.1% crystal violet solution for visualization, and images were acquired using a cell imaging system (Thermo Fisher, USA).

## Intracellular ATP Assay

The intracellular total ATP content was measured by the colorimetric method using an ATP assay kit (Abcam, UK) according to the manufacturer’s protocol. First, 1 × 10^6^ cells treated with the desired drugs were washed with cold PBS and collected by homogenization in assay buffer. Cell lysates were then clarified by centrifugation and subjected to deproteinization. Samples were loaded into 96-well plates for measurement. Colorimetric values were measured with a Multiskan MS microplate reader (Labsystems).

## Apoptosis Assay

A total of 2 × 10^5^ cells were seeded in 6-well plates one day before treatment. The cells were treated with compounds at the desired concentration and for the desired time. All cells and the culture medium were collected and washed with cold PBS. Induced cell death was quantified using the Pharmingen FITC Annexin V Staining Kit (BD Bioscience, USA). Apoptosis was analysed by flow cytometry using a NovoCyte Quanteon flow cytometer (Agilent).

## Reactive Oxygen Species (ROS) Measurement

Intracellular ROS levels were measured by staining with 5 µM DCFDA (Sigma‒Aldrich) for 15 min at room temperature. Samples were subjected to flow cytometry within 15 min. Mitochondrial ROS (mtROS) levels were measured by MitoSOX staining (Thermo Fisher, USA). Cells were stained with MitoSOX in HBSS for 15 min at 37°C. Data were collected using the NovoCyte Quanteon flow cytometer.

## Intracellular Free Fatty Acid Determination

The level of free fatty acids was determined by 4BODIPY™ 493/503 (4,4-difluoro-1,3,5,7,8-pentamethyl-4-bora-3a,4a-diaza-s-indacene) staining (Invitrogen). Cells were treated and then collected as previously mentioned. The cells were incubated in 2 μM BODIPY staining solution in PBS for 15 min at 37°C in the dark and were then subjected to flow cytometric analysis after washing.

## Immunofluorescence Staining

Animal tissues were fixed with 4% PFA and then embedded into paraffin blocks. The 5 μm thick sections were dewaxed and rehydrated in xylene and a series of ethanol solutions. Antigen retrieval was performed with 10 mM citrate buffer (Sigma‒Aldrich) at 96°C. Blocking was performed with 10% goat serum at room temperature for one hour. Tissue sections were incubated with an anti-cleaved caspase-3 (Cell Signaling technology, USA) or anti-Ki67 (Abcam, UK) antibody diluted 1:100 at 4°C overnight. The sections were then incubated with a secondary antibody (Invitrogen) and counterstained with DAPI for 10 min. Fluorescence images were acquired with an LSM 900 confocal microscope.

**Reference**

1. Tian J, Tang ZY, Ye SL, Liu YK, Lin ZY, Chen J, Xue Q: **New human hepatocellular carcinoma (HCC) cell line with highly metastatic potential (MHCC97) and its expressions of the factors associated with metastasis.** *Br J Cancer* 1999, **81:**814-821.

2. Dull T, Zufferey R, Kelly M, Mandel R, Nguyen M, Trono D, Naldini L: **A third-generation lentivirus vector with a conditional packaging system.** *J Virol* 1998, **72:**8463-8471.

3. Stewart SA, Dykxhoorn DM, Palliser D, Mizuno H, Yu EY, An DS, Sabatini DM, Chen IS, Hahn WC, Sharp PA: **Lentivirus-delivered stable gene silencing by RNAi in primary cells.** *RNA* 2003, **9:**493-501.

4. Gagliardi M, Matarazzo MR: **RIP: RNA Immunoprecipitation.** In *Polycomb Group Proteins: Methods and Protocols.* Edited by Lanzuolo C, Bodega B. New York, NY: Springer New York; 2016: 73-86
